# Potent therapeutic strategy in gastric cancer with microsatellite instability-high and/or deficient mismatch repair

**DOI:** 10.1007/s10120-024-01523-4

**Published:** 2024-06-26

**Authors:** Akira Ooki, Hiroki Osumi, Koichiro Yoshino, Kensei Yamaguchi

**Affiliations:** Department of Gastroenterological Chemotherapy, Cancer Institute Hospital of the Japanese Foundation for Cancer Research, 3-8-31 Ariake, Koto-Ku, Tokyo, 135-8550 Japan

**Keywords:** Gastric cancer, Microsatellite instability-high, Deficient mismatch repair, Immunotherapy, Chemotherapy

## Abstract

Gastric cancer (GC) is a common malignancy that presents challenges in patient care worldwide. The mismatch repair (MMR) system is a highly conserved DNA repair mechanism that protects genome integrity during replication. Deficient MMR (dMMR) results in an increased accumulation of genetic errors in microsatellite sequences, leading to the development of a microsatellite instability-high (MSI-H) phenotype. Most MSI-H/dMMR GCs arise sporadically, mainly due to MutL homolog 1 (*MLH1*) epigenetic silencing. Unlike microsatellite-stable (MSS)/proficient MMR (pMMR) GCs, MSI-H/dMMR GCs are relatively rare and represent a distinct subtype with genomic instability, a high somatic mutational burden, favorable immunogenicity, different responses to treatment, and prognosis. dMMR/MSI-H status is a robust predictive biomarker for treatment with immune checkpoint inhibitors (ICIs) due to high neoantigen load, prominent tumor-infiltrating lymphocytes, and programmed cell death ligand 1 (PD-L1) overexpression. However, a subset of MSI-H/dMMR GC patients does not benefit from immunotherapy, highlighting the need for further research into predictive biomarkers and resistance mechanisms. This review provides a comprehensive overview of the clinical, molecular, immunogenic, and therapeutic aspects of MSI-H/dMMR GC, with a focus on the impact of ICIs in immunotherapy and their potential as neoadjuvant therapies. Understanding the complexity and diversity of the molecular and immunological profiles of MSI-H/dMMR GC will drive the development of more effective therapeutic strategies and molecular targets for future precision medicine.

**Supplementary Information:**

The online version contains supplementary material available at 10.1007/s10120-024-01523-4.

## Introduction

Gastric cancer (GC) is the fifth most common cancer and the fourth leading cause of cancer-related deaths worldwide [1]. Based on comprehensive genomic analyses, GC has been demonstrated to be a heterogeneous disease composed of different subtypes, each with peculiar molecular aspects and specific clinical behavior. GC can be categorized into four subtypes according to The Cancer Genome Atlas (TCGA) molecular classification: microsatellite instability (MSI), chromosomal instability (CIN), Epstein–Barr virus (EBV)-positive, and genomically stable (GS) tumors [2]. Similarly, the Asian Cancer Research Group (ACRG) proposed the MSI subtype as one of four molecular subtypes with distinct molecular profiles and clinical outcomes [3]. The molecular classification of GC has paved the way for personalized therapies, among which the MSI subtype has gained significant attention. Microsatellites (MSs) are widespread, short, and repetitive DNA sequences throughout the human genome that are prone to DNA replication errors [4]. As the DNA mismatch repair (MMR) system plays a key role in recognizing and correcting these errors, the genetic and epigenetic inactivation of MMR genes leads to a deficient MMR (dMMR) system, resulting in a MSI-high (MSI-H) phenotype with genomic instability and a high tumor mutation burden (TMB) [5, 6]. Thus, considerable research effort has been invested in characterizing the genomic landscape of MSI-H/dMMR GC and identifying potential therapeutic targets for precision medicine.

The discovery of immune checkpoint inhibitors (ICIs) targeting programmed death-1 (PD-1) and programmed cell death ligand 1 (PD-L1) has led to a dramatic paradigm shift for cancer treatment, and MSI-H/dMMR is vulnerable to ICIs due to high immunogenicity and heavy infiltration of immune cells. Several pivotal trials have demonstrated that MSI-H/dMMR is significantly correlated with a response to ICIs across various types of tumor [7–9], indicating MSI-H/dMMR as an agnostic predictive biomarker for the efficacy of ICIs. In MSI-H/dMMR GC, treatment with ICI has shown promising and durable clinical responses, but a subset of patients still harbor intrinsic resistance [8–19]. The therapeutic paradigm will continue to evolve with an improved understanding of the immunological landscape in MSI-H/dMMR GC.

In this review, we summarize the biology, molecular and immunogenic landscape, and clinicopathological features, as well as the results of current chemotherapy and ICI treatment in MSI-H/dMMR GC. We also discuss potent therapeutic approaches in palliative and adjuvant settings, based on the state-of-the-art knowledge of MSI-H/dMMR GC from both basic and clinical viewpoints.

## Clinicopathological and molecular features of MSI-H/dMMR gastric cancer

**Fig. 1:**
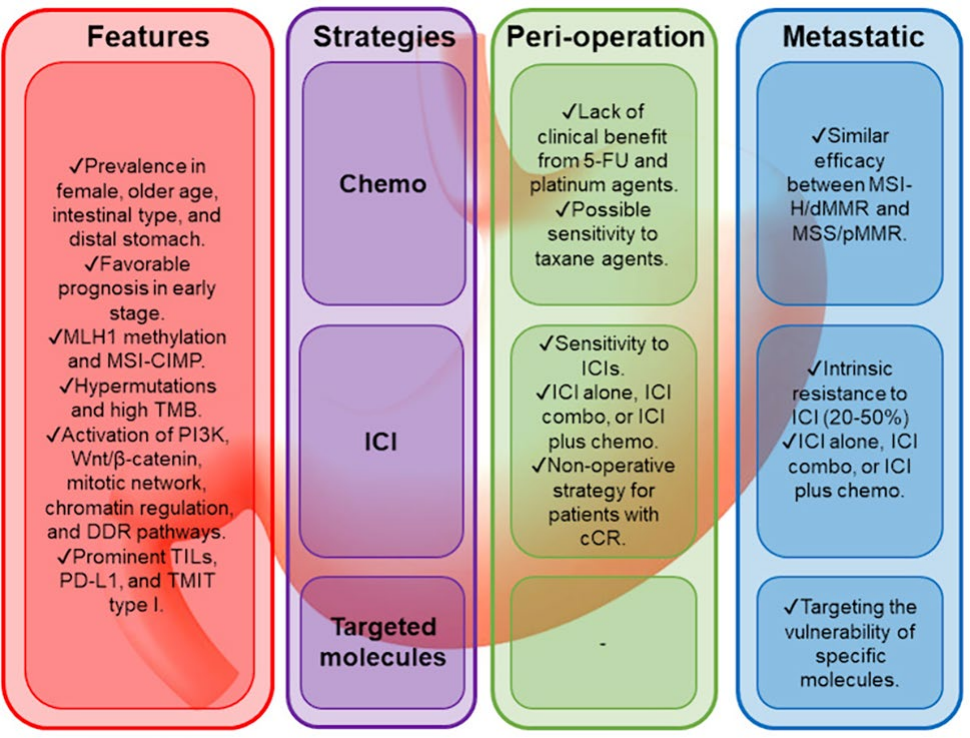
Characteristics and potent treatment of MSI-H/dMMR GC. CIMP, CpG island methylation phenotype; *TMB* tumor mutational burden, *DDR* DNA-damage response, *TILs* tumor-infiltrating lymphocytes, *TMIT* tumor microenvironment immune type, *ICI* immune checkpoint inhibitor, *cCR* clinical complete response

MSI-H/dMMR and microsatellite-stable (MSS)/proficient MMR (pMMR) GCs are distinct entities with widely differing genomic and immunogenic profiles and clinicopathologies (Fig. 1).

### Clinicopathological features of MSI-H/dMMR gastric cancer

The DNA MMR system is a highly conserved DNA repair mechanism and is composed of not only MutL homolog 1 (MLH1), MutS homolog 2 (MSH2), MutS homolog 6 (MSH6), and PMS1 homolog 2 (PMS2) but also mutL homologue 3 (MLH3), human mutS homologue 3 (MSH3), postmeiotic segregation increased 1 (PMS1), and exonuclease 1 (Exo1). In GC, mutations in MMR genes are relatively rare [20, 21], and MSI-H/dMMR phenotype is mostly developed from hypermethylation of CpG islands in the promoter region of the *MLH1* gene and subsequent epigenetic silencing of MLH1 expression [2, 22–28]. Molecular testing for MSI status and immunohistochemistry testing for MMR status are equally valid as initial screening approaches and have shown a high degree of concordance [29, 30]. The frequency of MSI-H/dMMR has been reported to range between 2.6 and 35.3% of GC (Table 1) [2, 3, 12, 13, 19, 21, 28, 30–78]. The prevalence of MSI-H/dMMR is determined to be tumor stage-dependent, as it is higher in the early stage (5.6–35.3%) than in the advanced stage (2.6–11.7%) GC [2, 3, 12, 19, 58, 71–73]. Lynch syndrome (LS), caused by germline inactivating mutations in the MMR genes, had a substantial risk for GC [21, 79]. Considering the limited association of family history with the MSI-H/dMMR phenotype [24, 59] and LS [79] and recognizing the implications for cancer surveillance and prevention in affected families, the National Comprehensive Cancer Network (NCCN) clinical practice guideline recommends universal testing for MSI or MMR for all newly diagnosed GC patients [80].

**Table 1:** Clinicopathological characteristics in MSI-H/dMMR GC patients

Characteristics	MSI-H/dMMR (compared to MSS/pMMR)	References
Incidence	3–35%	[2, 3, 12, 13, 19, 21, 28, 30–73, 75–78, 225]
Prevalence of Lynch syndrome	15%	[21]
Age	Elderly (median 63–73 years)	[2, 3, 28, 35, 37, 38, 41, 42, 47, 58, 59, 68–70, 81, 82]
Male/female ratio	Lower	[2, 38, 41, 46, 47, 57, 59, 70, 82]
Location of primary tumor	Antrum (middle or lower) > body	[3, 28, 37, 38, 41, 46, 47, 49, 54, 57, 59, 68, 70, 82]
Borrmann classification: type 4	Less	[20, 49, 58, 59, 68]
Lauren classification	Intestinal type > diffuse type	[3, 35–38, 41, 46, 47, 49, 54, 57–59, 66, 68, 70]
Tumor invasion	Less invasion	[66]
Nodal status	Less frequent	[3, 28, 35–37, 46, 47, 52, 57, 59, 66, 68]
Stage at diagnosis	Earlier	[3, 38, 41, 46, 47, 52, 54, 68, 75]
Chemosensitivity	Lower	[38, 83]
Recurrence rate	Less	[3]
Prognosis	Better	[35, 36, 41, 47, 49, 52, 53, 59, 66]
Histology	por1 and pap > tub2 and tub1	[82, 84]
Mucinous component	More	[36, 37, 58, 67, 85]
Tumor-infiltrating lymphocytes	More prominent	[16, 36, 86–97]
Stroma	Abundant tumor-associated inflammatory stroma with little or no desmoplastic stroma	[36, 85]
PD-L1 expression	Higher	[2, 10, 86, 88–90, 94, 96, 98, 99]
TMIT	Type I	[94–96]
TCGA molecular subtypes	MSI > CIN/GS/EBV	[2]

MSS group included those with either MSS or MSI-low status

por1, solid-type, poorly differentiated adenocarcinomas, *pap* papillary adenocarcinoma, *tub1* well-differentiated tubular adenocarcinoma, *tub2* moderately differentiated tubular adenocarcinoma, *TMIT* tumor microenvironment immune type, *TCGA* The Cancer Genome Atlas, *GS* genomically stable, *CIN* chromosomal instability, *EBV* Epstein–Barr virus

MSI-H/dMMR GC exhibits distinct clinicopathological entities compared to MSS/pMMR GC (Table 1) [2, 3, 10, 12, 13, 16, 19–22, 28, 30–73, 75–78, 81–99]. As loss of MMR function contributes to the development of tumor [20, 100], MSI-H/dMMR tumors share similar oncogenesis pathways and clinicopathological outcomes. In fact, MSI-H/dMMR GC typically exhibits predominant histopathological features of MSI-H/dMMR tumors, such as intestinal type, mucinous component, and highly pleomorphic tumor cells organized in papillary or solid-type poorly differentiated structures with prominent tumor-infiltrating lymphocytes (TILs) [20, 36, 37, 49, 58, 59, 67, 68, 82, 84, 85]. Clinically, MSI-H/dMMR GC is associated with female gender, shallower tumor invasion, early stages, and a lower number of lymph node metastases compared with MSS/pMMR GC, characteristics that are shared with MSI-H/dMMR colorectal cancer (CRC). [101, 102]. In contrast to MSI-H/dMMR CRC, MSI-H/dMMR GC has been associated with an older age of 65 years or more [2, 3, 28, 35, 37, 38, 41, 42, 47, 58, 59, 68–70, 81, 82]. The proportion of MSI-H/dMMR gradually increases with advancing age, accounting for 35–48% in GC patients over the age of 85 years [82, 103]. MSI-H/dMMR CRC predominantly affects the proximal colon [102], whereas MSI-H/dMMR GC is usually located in the distal stomach [3, 28, 37, 38, 41, 46, 47, 49, 54, 57, 59, 68, 70, 82].

The impact of prognosis on MSI-H/dMMR GC has mostly been evaluated by retrospective studies of resectable GC patients (Supplementary Table 1). Although conflicting results have been reported because of the limited numbers of patients, heterogeneous population with various disease stages, and different methodology for MSI/MMR detection, as well as the retrospective nature of the studies [38, 39, 58], resectable MSI-H/dMMR GC was generally associated with better prognosis than resectable MSS/pMMR GC [3, 28, 34–37, 41, 43, 44, 48–57, 59, 65–67, 70]. However, the prognostic value remains unclear in a setting with metastatic and recurrent unresectable GC [45, 47, 87, 104].

### Molecular features of MSI-H/dMMR gastric cancer

MSI-H GC exhibits concurrent hypermethylation of multiple tumor suppressor genes, characterizing MSI-CpG island methylation phenotype (CIMP) [105]. Notably, MLH1 hypermethylation is specific to MSI-CIMP. Genetic instability caused by MSI-H/dMMR leads to the accumulation of thousands of mutations and single-nucleotide variants (SNVs) through processes such as DNA polymerase slippage and unequal crossing over [16, 106, 107], creating a hypermutator phenotype [2, 3, 23]. The MSI-H genomes are predominantly enriched for frameshift insertions or deletions (indels) but not copy number alterations [16, 106]. These genomic alterations mainly occur in MS-bearing genes and affect both coding and non-coding regions. Indels in coding genes result in frameshift mutations, leading to truncated proteins with impaired or no function. The progressive accumulation of diverse random mutations, followed by clonal selection, results in the emergence of a dominant clone displaying heightened aggressive behaviors. This evolutionary process leads to the development of an entire dysplastic lesion, referred to as field cancerization [108–110].

Developments in high-throughput genomic technologies have led to a better understanding of the molecular profiles in MSI-H/dMMR GC (Table 2) [2, 3, 16, 22, 23, 32–34, 44, 67, 68, 90, 92, 104, 107, 111–134]. Recurrent MSI has been frequently observed in specific gene clusters spanning 23 tumor types, including immune response, DNA-damage response (DDR), chromatin regulation, and transforming growth factor beta (TGF-β) [32]. MSI-H/dMMR GC shows frequent dysregulation of signaling pathways, including the phosphatidylinositol-4,5-bisphosphate 3-kinase (PI3K)/phosphatase and tensin homolog (PTEN)/mammalian target of rapamycin (mTOR), the Wnt/β-catenin, mitotic network, chromatin regulation, DDR, and MMRs [2, 3, 20, 23, 67, 92, 107, 133]. Compared to MSS/pMMR GC, MSI-H/dMMR GC displays a higher frequency of mutations of TGF-β receptor 2 (*TGFBR2*), activin A receptor type 2A (*ACVR2A*), AT-rich interaction domain 1A (*ARID1A*), phosphatidylinositol-4,5-bisphosphate 3-kinase catalytic subunit alpha (*PIK3CA*), insulin-like growth factor 2 receptor (*IGF2R*), BCL2 associated X, apoptosis regulator (*BAX*), histone modifying factor, lysine methyltransferase 2C (*KMT2C*), lysine methyltransferase 2D (*KMT2D*), ring finger protein 43 (*RNF43*), PR/SET Domain 2 (*PRDM2*), and E2F transcription factor 4 (*E2F4*) genes but a lower incidence of *TP53* mutations [2, 3, 22, 34, 68, 90, 92, 107, 111, 117, 125, 135]. Of note, an analysis of 5,930 cancer exomes from the TCGA database identified MSI loci with high instability in specific tumor types, leading to tumor-specific instability signatures [33]. For instance, *BAX*, *PRDM2*, mediator complex subunit 1 (*MED1*), and *KMT2C* are enriched in MSI-H/dMMR GC [32, 125]. In contrast, the B-Raf proto-oncogene, serine/threonine kinase (*BRAF*) mutation and neurotrophic tyrosine receptor kinase (*NTRK*) gene fusion have been identified as prevalent in MSI-H/dMMR CRC, whereas these alterations are not enriched in MSI-H/dMMR GC [2, 32, 136]. Thus, impairment of the MMR system drives oncogenic deregulation in both cancer-specific and MSI-specific ways. Importantly, parallel evolution of subclonal driver mutations occurs in *RAS*, *PIK3CA*, switch/sucrose nonfermentable (SWI/SNF)-complex genes, and immune evasion regulators. The MSI hypermutator phenotype remains active during cancer progression, generating more subclonal mutations and, consequently, extreme intratumoral heterogeneity.

**Table 2:** Dysregulated genetic alterations in MSI-H/dMMR GC

Molecules	Signaling pathway	MSI	MSS	Special comments
		Frequency of genetic aberrations (%)		
Cell cycle				
TP53	Regulator of cell cycle	17–67	41–78	MSI-H/dMMR GC exhibits a lower incidence of *TP53* mutations than MSS/pMMR GC. E2F4, a transcription factor regulating cell cycle progression, shows frequent alterations in MSI-H/dMMR GC, possibly promoting tumor growth [22, 34, 68]. *E2F4* frameshift mutations often coincide with *MSH3* mutations, indicating a secondary mutator effect [226]. Interaction between E2F4 and the RB transcriptional corepressor 1 (Rb1) family can induce cell differentiation and growth arrest at G1 phase [227], with frameshift mutations in *E2F4*, possibly reducing the differentiation-promoting E2F4-Rb1 complex [226]
MDM2	Regulator of cell cycle	2–5	0	
CDKN2A	Regulator of cell cycle	0–22	3–18	
CCND1	Regulator of cell cycle	0–15	3–5	
CCNE1	Regulator of cell cycle	0–5	3–40	
E2F4	Transcription factor	36–61	0	
RTK/RAS/MAPK and PI3K				
BRAF	MAPK/PI3K pathway	0–12	0–7	Although MSI-H/dMMR GCs are enriched with *KRAS* mutations, they generally lack targetable amplifications of *HER2* and are not associated with *BRAF V600E* mutations [2, 28, 118, 221]. The high prevalence of the *KRAS* mutation in MSI-H/dMMR GC suggests that the activation of KRAS-dependent pathways contributes to the tumorigenesis of MSI-H/dMMR GC [221]. Although no pathogenic mutations were found in the hotspot regions of epidermal growth factor receptor (*EGFR*), deletions in the 3′- untranslated region (UTR) polyA repeat were found in a high proportion of MSI-H/dMMR GC [119]. Mutations in the 3′-UTR polyA repeat of *EGFR* have been found to be associated with EGFR overexpression in CRC through the enhancement of EGFR mRNA stability [228], suggesting a putative role for these mutations in MSI-H/dMMR GC development. The *IGF2R* gene, which belongs to the insulin growth receptor family, is considered pivotal in GC progression. It binds to the latent complex of TGFB1 and activates TGFB1, sequentially implicating tumorigenesis [229]. MSI-H/dMMR GCs frequently exhibit mutations in the *IGF2R* gene controlling proliferation [22, 67, 124, 229]. *PTEN* loss-of-function mutations in MSI-H/dMMR tumors was associated with PI3K/Akt/mTOR pathway enrichment, an immunosuppressive TME with depletion of CD8+ T cells, and an abundance of tumor-associated macrophages, resulting in a negative response to PD-1 blockade in 45 patients with MSI-H/dMMR gastrointestinal tumors, including 18 GC patients [180]. Similarly, mutated genes in the PI3K/Akt/mTOR pathway were negatively correlated with densities of CD3+, CD8+, and FOXP3+ TILs and the transcription of immune-related genes [92]. The PI3K/Akt/mTOR pathway may be implicated in the potential primary resistance to ICIs in MSI-H/dMMR GC
KRAS	MAPK/PI3K pathway	12–33	1–24	
PIK3CA	PI3K pathway	33–60	3–19	
PIK3R1	PI3K pathway	15–60	0–10	
mTOR	PI3K pathway	14–40	3–8	
TSC2	PI3K pathway	40	0–8	
PTEN	PI3K pathway	11–33	1–10	
EGFR	RTK	0–5	1–12	
ERBB2	RTK	5–47	0–25	
ERBB3	RTK	14–33	5–7	
FGFR2	RTK	0–2	0–9	
FGFR1	RTK	0	3–5	
IGF2R	RTK	19–33	0–7	
MET	RTK	0–3	0–12	
ALK	RTK	16–33	5	
VEGFA	Angiogenesis	0	3–7	
Cell adhesion and proliferation				
CTNNB1	Wnt/β-catenin pathway	0–11	3–11	Wnt signaling plays a crucial role in gastrointestinal tract development and is implicated in the pathogenesis of GC [230]. Catenin beta 1 (*CTNNB1*) encodes β-catenin, an essential component of this pathway. β-Catenin promotes adherens junction formation by binding to E-cadherin, but also influences cell proliferation, differentiation, immune evasion, and epithelial-mesenchymal transition [16, 231]. Although *CTNNB1* genetic aberrations are rare, MMR deficiency often leads to indel mutations in Wnt/β-catenin pathway-related genes, particularly *RNF43*, in MSI-H/dMMR GC [2, 23, 90, 92, 107, 117, 133, 232]. RNF43, a transmembrane ubiquitin E3 ligase, negatively regulates Wnt signaling by ubiquitinating Frizzled receptors for degradation [233]. In addition, long non-coding RNA MIR99AHG activates this pathway in MSI-H/dMMR GC, acting as a competitive endogenous RNA that is a ubiquitous regulatory network in the human genome [234]. In an analysis of whole-exome sequencing and single-cell RNA-seq using tissue samples from the phase II trial of pembrolizumab in MSI-H GC, tumors with activated Wnt/β-catenin pathway as well as a lower TMB, genomic features of the GS subtype in TCGA, and an abundance of cancer-associated fibroblasts (CAFs) and terminally differentiated exhausted CD8+ T cells, were implicated in pembrolizumab-insensitive [16]. Thus, aberrant activation of Wnt/β-catenin pathway may drive carcinogenesis, tumor progression, and immune resistance. Frequent mutations in *ACVR2A* and *TGFBR2* lead to downregulation of the TGF-β signaling pathway in most MSI-H/dMMR GCs. However, the transcriptomic analysis of MSI-H/dMMR gastrointestinal tumors, including GS, showed more enriched immunosuppressive TME with upregulation of TGF-β pathway in non-responders to PD-1 blockade [179]
APC	Wnt/β-catenin pathway	0–28	4–13	
RNF43	Wnt/β-catenin pathway	35–80	0–5	
CDH1	Cell adhesion	5–40	4–14	
MYC	Transcription factor	0–20	14–32	
SMAD4	TGFβ pathway	0–5	1–9	
TGFBR2	TGFβ pathway	61–90	0–9	
ACVR2A	TGFβ pathway	28–86	0–12	
Cell apoptosis				
BAX	Apoptosis	14–61	0–7	MSI-H/dMMR GC frequently exhibits mutations in the *BAX* gene, which are involved in cell cycle regulation and apoptosis. *BAX* mutations, along with alterations in other genes, contribute to genetic instability and the development of a malignant phenotype [20, 22, 34, 67, 68, 124, 125]. These findings suggest that BAX plays a significant role in the pathogenesis of MSI-H/dMMR GC
Chromatin regulation				
KMT2D	Histone modifiers	77	17	Both SWI/SNF chromatin remodeling and histone modification are major processes of chromatin regulation extensively altered in MSI-H/dMMR GC [90, 92, 96, 105–107, 114, 116, 117, 127, 128, 133, 235]. The SWI/SNF complex consists of core subunits and variant subunits, including ARID1A, and ATPase, including SWI/SNF related, matrix associated, actin dependent regulator of chromatin, subfamily a, member 4 (SMARCA4) [106, 133, 236]. SWI/SNF is the most frequently mutated chromatin-regulatory complex in human cancer, with approximately 20% of all human cancers harboring mutations affecting the SWI/SNF complex [236]. SWI/SNF complex aberrations are not only important for carcinogenesis but may also contribute to cancer progression [23]. ARID1A is a key component of the SWI/SNF chromatin remodeling complex and is crucial for regulating various cellular processes, including development, differentiation, proliferation, and DNA repair [114, 117, 209]. In a pan-cancer analysis of NGS data from 4591 cases, ARID1A was the most frequently altered SWI/SNF gene associated with a higher TMB and MSI-H status [235]. In GC, ARID1A is preferentially mutated in MSI and EBV subtypes, resulting in the loss of its expression [2, 117]. Moreover, the prevalence of the ARID1A mutation did not significantly differ between early and advanced MSI-H/dMMR GCs [106]. ARID1A was shown to recruit the MMR protein MSH2 to chromatin during DNA replication and to promote MMR activation [237]. Mechanistically, ARID1A mutations lead to dysregulation of transcription, DDR, the MMR system, DNA repair, and chromatin segregation, thereby promoting tumorigenesis through compromised diverse gene programs and cellular processes [211, 215]. Other SWI/SNF complex gene mutations, such as SMARCA4, are also frequently mutated in MSI-H/dMMR GC. As a histone modification, KMT2C and KMT2D are critical for RNA polymerase II-dependent transcription and are embedded in a complex of proteins associated with the SET1 (COMPASS) family that mono-, di-, and tri-methylate histone 3 lysine 4 at transcription enhancers throughout the human genome [238]. Mutations of *KMT2D* and *KMT2C* may disrupt transcriptional enhancers, transcription factor-dependent programs, and DNA repair processes, affecting tumor suppression and immune evasion [239]. The high frequency of *KMT2C* and *KMT2D* mutations often co-occurs with driver mutations, such as *TP53*, *PIK3CA*, *PTEN*, and the SWI/SNF complex component *ARID1A* mutation, suggesting they could be early tumorigenic events and alter the epigenomic landscape to permit additional oncogenic changes [240]. Furthermore, PRDM2 is a histone methyltransferase considered a tumor suppressor gene, as PRDM2 can induce G2-M arrest and apoptosis in MSI-H CRC [241]. These findings underscore the significance of chromatin regulation alterations in MSI-H/dMMR GC pathogenesis
KMT2C	Histone modifiers	47	11	
PRDM2	Histone modifiers	19–57	0–9	
ARID1A	SWI/SNF	40–84	5–28	
SMARCA4	SWI/SNF	20–43	3–8	
DNA-damage response				
ATR	DNA repair systems	21–33	0	DDR pathways play a critical role in genomic integrity through the activation of DNA repair signaling and their interaction with cell cycle checkpoints [242]. The MMR system corrects the errors that might occur during DNA replication, whereas homologous recombination and non-homologous end joining are involved in the repair of DSBs. Dysregulation of the DDR pathway is associated with predisposition to cancer development [243]. Although ataxia-telangiectasia mutated (ATM) and ataxia telangiectasia and RAD3-related (ATR) appear to phosphorylate many of the same cellular substrates, they generally respond to distinct types of DNA damage. ATM is the primary mediator of the response to DNA DSBs that can arise by exposure to ionizing radiation. ATM signals the cell cycle checkpoint to slow cell passage through the cycle to aid in DNA repair [244]. By contrast, ATR plays only a backup role in the DSB response, although it directs the principal response to ultraviolet damage and stalls in DNA replication. ATR not only stabilizes replication forks but also activates the G2/M checkpoint [129]. MED1 is a central component of the mediator complex that acts as a molecular bridge between RNA polymerase and various transcription activators [245]. *PRKDC* encodes a catalytic subunit of DNA-dependent protein kinase (DNA-PK) that acts as a pivotal component of DSB repair and recombination, serving to maintain genomic stability [246]. MSI-H/dMMR GC commonly exhibits dysregulation of DDR pathway components, including ATM, ATR, MED1, and PRKDC, which may contribute to the development of gastric malignancies with impaired MMR function [2, 67, 125, 129, 130, 247, 248]
ATM	DNA repair systems	41–43	8	
MED1	DNA repair systems	19–43	0	
MSH3	DNA repair systems	17–56	0–14	
MSH6	DNA repair systems	18–43	0–9	
PRKDC	DNA repair systems	24–42	4	
BRCA2	DNA repair systems	0–60	0–8	
Immune system				
JAK1	IFN pathway	15	1–5	Recurrent frameshift mutations in Janus kinase 1/2 (*JAK1/2*) and β2-microglobulin (*β2M*) genes often occur in MSI-H/dMMR tumors compared to MSS/pMMR tumors. The JAK family is expressed in immune, stromal, and tumor cells and mediates PD-L1 expression and IFN-γ signaling. *JAK1/2* loss-of-function mutations have been reported as a genetic mechanism of primary resistance to ICI treatment in melanoma and MSI-H/dMMR CRC [131, 249, 250]. In GC, recurrent frameshift mutations in *JAK1/2* results in reduced expression of IFN-γ signaling and multiple antitumor immune signatures [16, 131]. In contrast, responders to ICIs have been found to have aberrant activity of IFN-γ signaling [179]. Thus, the suppressed activity of the IFN-γ signaling has been implicated in resistance to ICI treatment. The *β2M* gene is a crucial component of trimeric MHC class I molecules [251]. Frameshift mutations were observed in the cMS regions of the *β2M* gene in MSI-H GC [34, 104, 133], leading to the complete loss of MHC class I expression [252]. B2M loss has been associated with resistance to ICIs in melanoma [253], while *B2M* mutations may not necessarily preclude a response to ICI treatment in MSI-H GC [254], similar to MSI-H CRC [104]. There are likely organ-specific traits influencing the response to ICIs even in MSI-H tumors. TMB, defined as the total number of mutations per coding area of a tumor genome, emerges as a promising predictive biomarker for responding to anti-PD-1/PD-L1 inhibitors in GC [134, 255, 256]. Notably, even in MSI-H GC, the median TMB value was significantly higher in responders to anti-PD-1 Ab than in non-responders [16]. Among populations with high TMB, indel mutational load and clonal mutation burden are likely to serve as more stringent predictors of immunotherapy outcomes [138, 176, 177]. Cancer immunoediting is likely to serve as a crucial mechanism of resistance to immunotherapy [142]. MSI-H GC frequently harbors mutations that interfere with MHC class I antigen presentation [2], which can effectively mitigate selective pressure because MHC class I molecules play a pivotal role in orchestrating immune responses [23]. Furthermore, the loss of human leukocyte antigen (HLA)-A and the mutation of HLA-B are also more frequent in MSI-H GC compared to MSS GC [2, 178]
JAK2	IFN pathway	5–13	1	
B2M	MHC	21–44	0	
TMB	Neoantigen	24–30	3	

The frequency of each genetic alteration is referenced from several studies [2, 3, 16, 22, 23, 32–34, 44, 67, 68, 90, 92, 104, 107, 111–134]. Refer also to the cBioPortal for Cancer Genomics (https://www.cbioportal.org/

### Immunological features of MSI-H/dMMR gastric cancer

The heightened occurrence of nonsynonymous SNVs and frameshift mutations generates numerous immunogenic neoantigens on the major histocompatibility complex (MHC) class I molecules on tumor cells and on MHC class I and II on antigen-presenting cells, thereby priming T cells to identify them as non-self and recruiting T cells within the tumor as TILs [11, 137, 138]. In fact, MSI-H GCs are characterized by prominent TILs [16, 36, 86–97]. In addition, MSI-H/dMMR GCs exhibit TILs containing abundant M1 macrophages and natural killer (NK) cells [139–141], along with activated CD8+ cytotoxic T-lymphocytes and T helper type 1 cells characterized by interferon-gamma (IFN-γ) response [16, 86, 89, 90, 92, 93, 95]. A transcriptome and RNA sequencing (RNA-seq) analysis revealed enrichment of pathways related to immune cell signaling, cytotoxicity, NK cell function, and antigen processing in MSI-H GC [2, 86]. Consequently, in the early stage, activated immunosurveillance may contribute to favorable prognostic outcomes in patients with MSI-H/dMMR GC compared to those with MSS/pMMR GC [3, 91, 93]. However, tumor elimination or immune surveillance is not always efficient. Cancer immunoediting is profoundly influenced by immune selective pressure stemming from the high immunogenicity of MSI-H/dMMR tumors, ultimately leading to immune escape [142]. Of note, MSI-H/dMMR tumors also stimulate the expression of inhibitory immune checkpoint molecules, including PD-1 and PD-L1. The PD-L1/PD-1 signaling axis creates an immune-evasive state in the tumor microenvironment (TME) [6]. In GC, PD-L1 is more frequently overexpressed on both tumor cells and immune cells in MSI-H/dMMR tumors compared to MSS/pMMR tumors [2, 10, 86, 88–90, 94, 96, 98, 99]. Based on their PD-L1 expression and the number of TILs, tumors can be classified into four subtypes according to the tumor microenvironment immune type (TMIT), which predicts suitable candidates for immunotherapy [143]. An RNA analysis of 414 GC samples in the pan-cancer database of TCGA revealed that approximately 70% of MSI-H/dMMR GCs exhibited TMIT type I, characterized by high PD-L1 and CD8A expression [95], driving adaptive immune resistance. Therefore, PD-1/PD-L1 blockade may reverse the immune-evasive state into an antitumor response state, providing a rationale for treating MSI-H/dMMR GC patients with ICIs targeting PD-1/PD-L1.

## Treatment of patients with MSI-H/dMMR gastric cancer

In this section, the clinical efficacy of chemotherapy agents for patients with MSI-H/dMMR GC is described (Fig. 1).

### Cytotoxic chemotherapy

As the MMR system not only repairs DNA replication errors but also activates signaling pathways that trigger apoptosis in response to DNA damage, impairment of the MMR system may be a relevant mechanism of resistance to a variety of cytotoxic agents, including 5-fluorouracil (5-FU) and cisplatin [83, 144–149]. In CRC, MSI-H/dMMR tumors are associated with a favorable prognosis and lack of efficacy of adjuvant fluoropyrimidine monotherapy [62, 101, 150], as well as little benefit from neoadjuvant fluoropyrimidine-based chemotherapy [151], suggesting consideration of MSI/MMR status in treatment decision-making. The impact of cytotoxic chemotherapy on perioperative settings, including adjuvant chemotherapy, for MSI-H/dMMR GC patients has been evaluated in several exploratory analyses and retrospective studies (Supplementary Table 1).

Patients with MSI-H/dMMR GC might have limited benefits from neoadjuvant chemotherapy [30, 60–62, 65, 70]. In addition, MSI-H/dMMR tumors exhibited low pathological tumor response to neoadjuvant chemotherapy [30, 37, 61, 62, 64, 152, 153]. Similarly, many retrospective studies have reported no treatment benefit from 5-FU-based adjuvant chemotherapy [38–40, 43, 46, 47, 50–52, 56, 154], despite some conflicting results [42, 44, 54, 55]. In terms of oxaliplatin (L-OHP) among platinum compounds, the addition of L-OHP to adjuvant treatment with 5-FU demonstrated prolonged survival compared to those with 5-FU alone for patients with stage III MSI-H/dMMR CRC [155]. In GC, a post hoc analysis was performed according to the MSI status in a phase III CLASSIC trial that demonstrated the survival benefit of adjuvant CAPOX (capecitabine plus L-OHP) chemotherapy over surgery alone for patients with stage II and III GC [50]. Adjuvant CAPOX chemotherapy failed to demonstrate an improvement in disease-free survival (DFS) for patients with MSI-H, in contrast to those with MSS. In a meta-analysis of perioperative chemotherapy using only randomized individual patient data from four-phase III resectable GC trials [52], chemotherapy plus surgery showed significantly prolonged survival compared to surgery alone in patients with MSS but not in those with MSI-H. It remains unclear whether taxanes, such as docetaxel and paclitaxel, are effective against MSI-H/dMMR GC tumors [74, 156, 157].

Collectively, previous studies have challenged the clinical benefits of perioperative chemotherapy in MSI-H/dMMR patients due to their favorable prognosis and the limited efficacy of chemotherapy, raising the possibility of avoiding unnecessary chemotherapy for patients with resectable MSI-H/dMMR GC, especially for older patients with early-stage disease. However, the routine clinical use of MSI/MMR status in therapeutic decision-making for perioperative chemotherapy, including adjuvant chemotherapy, is still debated due to limited and retrospective data [158–160], emphasizing the need for large prospective trials based on MSI status. In a palliative setting, exploratory analyses of clinical trials showed that chemotherapy, including 5-FU plus platinum or paclitaxel, has equivalent outcomes in patients with MSI-H/dMMR or MSS/pMMR GC (Supplementary Table 1).

### Immune therapy

ICIs targeting PD-1 have dramatically changed therapeutic paradigms due to the durable clinical response in GC [8–19, 76, 161]. However, the efficacy of ICI monotherapy is limited to certain patient populations [80, 158, 162–164]. Building on the clinical benefits observed with anti-PD-1 antibody (Ab) pembrolizumab in MSI-H/dMMR tumors across various organ sites [7–9, 11], the Food and Drug Administration granted the first tumor-agnostic approval for pembrolizumab in May 2017 for MSI-H/dMMR tumors. The clinical efficacy of PD-1/PD-L1 Ab for patients with MSI-H/dMMR GC in pivotal trials is summarized in Table 3 [8, 9, 11–19, 71, 76, 165–167].

**Table 3:** Studies of immune checkpoint inhibitors for MSI-H/dMMR GC patients

Trials	Arm/cohort	Agents	Phase	Line	MSI status	No of pts	ORR (%)	DCR (%)	PFS, median (95% CI), months	OS, median (95% CI), months	HR for OS (95% CI)	OS rate at 12 months (%)	OS rate at 24 months (%)	Reference
CheckMate-649	Nivo + chemo arm	Nivo+chemo	III	1st	MSI-H	23	55	–	–	38.7 (8.4–44.8)	0.38 (0.17–0.84)	–	–	[71]
					MSS	696	59		–	13.8 (12.4–14.5)	0.78 (0.70–0.88)	–	–	
KEYNOTE-859	Pembro + chemo arm	Pembro + chemo	III	1st	MSI-H	39	79.5	–	–	–	0.34 (0.18–0.66)	–	–	[76]
					MSS	641	49.8	–	–	–	0.79 (0.70–0.89)	–	–	
KEYNOTE-062	Pembro arm	Pembro	III	1st	MSI-H	14	57.1	78.5	11.2 (1.5–NR)	NR (10.7–NR)	0.29 (0.11–0.81)	79	71	[12, 165]
					MSS	242	12.4	–	1.7 (1.5–2.6)	9.5 (7.2–12.4)	–	45.0	27	
	Pembro + chemo arm	Pembro + chemo			MSI-H	17	64.7	82.3	NR (3.6–NR)	NR (3.6–NR)	0.37 (0.14, 0.97)	71	65	
					MSS	240	47.5	–	6.9 (5.7–7.3)	12.3 (10.5–13.3)	–	51.7	24	
KEYNOTE-012	Cohort of GC with PD-L1+	Pembro	Ib	≥ 1st	MSI-H	4	50	50	–	–	–	–	–	[166]
KEYNOTE-061	Pembro arm	Pembro	III	2nd	MSI-H	15	46.7	86.7	17.8 (2.7–NR)	NR (5.6–NR)	0.42 (0.13–1.31)	73	59	[12, 167]
					MSS	281	9.3	–	1.5 (1.4–1.6)	6.5 (5.0–8.6)	–	32.0	–	
KEYNOTE-016	Cohort of GC	Pembro	II	≥ 2nd	MSI-H	5	60	60	–	–	–	–	–	[11]
KEYNOTE-158	Cohort of GC	Pembro	II	≥ 2nd	MSI-H	42	31.0	47.7	3.2 (2.1–12.9)	11.0 (5.8–31.5)	–	–	–	[8, 9]
KEYNOTE-059	Cohort-1	Pembro	II	≥ 3rd	MSI-H	7	57.1	71.4	NR (1.1–NR)	NR (1.1–NR)	–	71	57	[12, 13]
					MSS	167	10.3	22.2	2.0 (1.9–2.0)	5.4 (4.1–6.5)	–	23 (25)	13	
CheckMate-032	Nivo monotherapy	Nivo	I/II	≥ 2nd	MSI-H	7	29	71	–	14.8 (1.5–NR)	–	57	29 (at 18 months)	[14, 15]
					MSS	18	11	28	–	6.5 (3.0–12.4)	–	33	17 (at 18 months)	
NCT02589496	Cohort B	Pembro	II	≥ 2nd	MSI-H	19	55.6	88.9	26.9	26.9	–	–	–	[16]
GARNET	Cohort F	Dostarli	I	≥ 2nd	MSI-H	8	37.5	–	–	–	–	–	–	[17]
NCT03667170	Cohort of GC	Envafolimab	II	≥ 2nd	MSI-H	18	44.4	83.3	NR (11.1–NR)	NR (NR–NR)	–	83.3	–	[18]

When there is no data available for the MSS population, data from all patients, including MSS and MSI-H, were presented

*No of pts* number of patients, *ORR* overall response rate, *DCR* disease control rate, *PFS* progression-free survival, *OS* overall survival, *HR* hazard ratio, *Nivo* nivolumab, *Pembro* pembrolizumab, *Dostarli* dostarlimab, *NR* not reached

#### Palliative immune therapy

Although ICI monotherapy demonstrated no improvement in overall survival (OS) compared to standard chemotherapy in the overall population in the phase III KEYNOTE-062 and KEYNOTE-061 trials [165, 167], among patients with MSI-H/dMMR GC, ICI monotherapy consistently showed a superior survival curve compared to chemotherapy from the beginning of treatment, accompanied by higher overall response rate (ORR) and prolonged progression-free survival (PFS) [12]. The positive effects of PD-1/PD-L1 Ab for patients with MSI-H/dMMR GC have been confirmed in several systematic reviews and meta-analyses [72, 73, 168–171]. Thus, the MSI-H/dMMR status serves as a promising molecular hallmark, indicating potential sensitivity to ICI treatment, with an ORR ranging from 29 to 60% and a disease control rate (DCR) ranging from 48 to 89% (Table 3) [8, 9, 11–19, 71, 76, 165–167]. Recently, the clinical benefits of anti-PD-1 Ab in combination with first-line chemotherapy for patients with the human epidermal growth factor receptor 2 (HER2)-negative GC were demonstrated in pivotal phase III trials [71, 76, 161, 172–174]. A systematic review and meta-analysis were conducted to evaluate the treatment efficacy for 3355 GC patients using five randomized phase III trials of the addition of anti-PD-1 Ab to first-line cytotoxic chemotherapy [168]. The estimated HR of OS in the combination of anti-PD-1 Ab and chemotherapy versus chemotherapy alone was significantly improved in MSI-H patients (HR, 0.38; 95% CI 0.20–0.70) compared to MSS patients (HR, 0.78; 95% CI 0.70–0.87).

For MSI-H/dMMR tumors, the optimal treatment regimen, whether ICI monotherapy or ICI combined with chemotherapy, remains uncertain. Chemotherapy has been demonstrated to induce immunogenic cell death in tumor cells, triggering recognition by dendritic cells (DCs) and the activation of CD8+ T cells [175]. Consequently, combining ICI with chemotherapy may offer a promising approach to overcoming primary resistance to immunotherapy. In the phase III KEYNOTE-062 trial, among patients with MSI-H tumors and a PD-L1 combined positive score of 1 or greater, pembrolizumab monotherapy demonstrated a trend toward a more favorable OS compared to chemotherapy alone (HR, 0.29; 95% CI 0.11–0.81), while pembrolizumab plus chemotherapy showed a slightly lower efficacy (HR, 0.37; 95% CI 0.14–0.97) [12, 165]. Conversely, pembrolizumab plus chemotherapy showed superior ORR and PFS compared to pembrolizumab monotherapy, with an ORR of 64.7% and estimated HR for PFS of 0.45 (95% CI 0.18–1.11) versus an ORR of 57.1% and HR for PFS of 0.72 (95% CI 0.31–1.68), respectively. Although this trial was not intended to directly compare pembrolizumab alone and pembrolizumab in combination with chemotherapy, the results suggest a potential benefit for MSI-H/dMMR patients when receiving a combination of chemotherapy on multiple occasions to induce an initial response. However, prolonged administration of chemotherapy may not provide additional benefits. Based on the hypothesis that MSI-H/dMMR patients might benefit from a short course of chemotherapy, an ongoing phase II AuspiCiOus proof-of-principle trial aims to assess the treatment efficacy of a sequential method involving two cycles of CAPOX chemotherapy, followed by monotherapy with the anti-PD-1 Ab retifanlimab (NCT05177133).

A relevant proportion of MSI-H/dMMR GC patients undergoing anti-PD-1/PD-L1 monotherapy still exhibit intrinsic resistance, with a progressive disease rate of 20–50% in clinical trials (Table 3), indicating that MSI-H/dMMR GCs still display substantial heterogeneity from an immunological viewpoint. Molecular analyses of MSI-H/dMMR GC underscore alterations in genes that regulate the antigen-presenting machinery, IFN-γ signaling, Wnt/β-catenin pathway, TGF-β pathway, and PI3K pathway, contributing to resistance to ICI treatment (Table 2) [2, 16, 92, 131, 138, 176–180]. In addition, a comprehensive assessment of various components within the TME will be crucial for a more accurate prediction of the success or failure of ICI treatment in patients with MSI-H GC [16, 181, 182]. Thus, elucidating the determinant mechanisms of immunotherapy sensitivity and resistance would pave the way for the development of new treatment strategies.

## Potent therapeutic strategies in MSI-H/dMMR gastric cancer

**Fig. 2:**
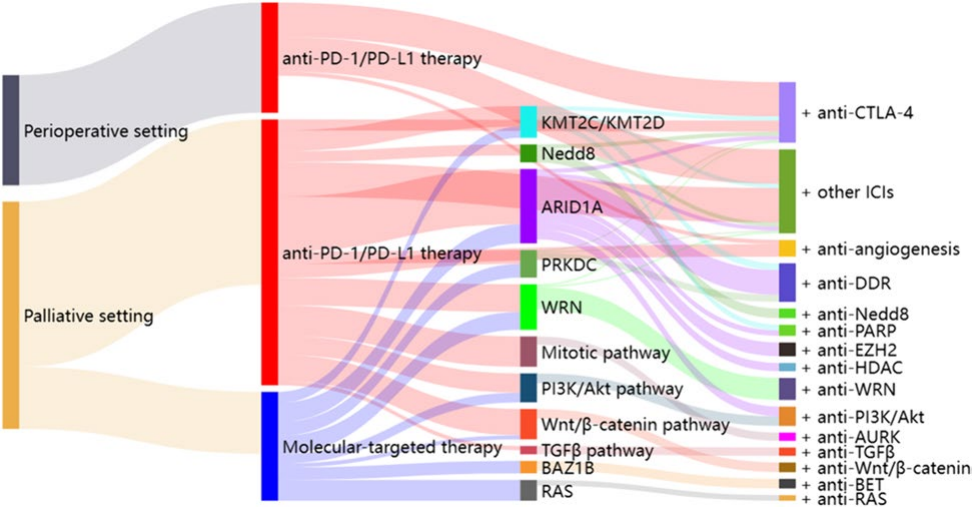
Summary of potent therapeutic strategies for MSI-H/dMMR GC. In the perioperative setting, neoadjuvant or perioperative ICI therapy has already become one of the options for patients with MSI-H/dMMR GC in the NCCN guidelines. Besides monotherapy with ICIs, combining anti-PD-1/PD-L1 Ab with anti-CTLA-4 Ab or other ICIs, including TIGIT, TIM3, and LAG-3 inhibitors, as well as an OX40 agonist Ab, are also considered. In the metastatic setting, potent strategies include dual immune checkpoint blockade, combining anti-PD-1/PD-L1 Ab with molecular targeted agents, and targeting vulnerabilities with selective molecule inhibition. A dual immune checkpoint blockade include combination of anti-PD-1/PD-L1 Ab with anti-CTLA-4 Ab or other ICIs. Mutations of *PRKDC*, *KMT2D*, and *KMT2C* gene mutations potentially serve as predictive biomarkers for immunotherapy response. Effective partners for ICIs include angiogenesis inhibitors such as apatinib and lenvatinib, Nedd8-activating enzyme inhibitors, and DDR inhibitors for ARID1A deficiency. Promising therapeutic targets for MSI-H/dMMR tumors include inhibitors of WRN, TGF-β, PI3K/Akt, Wnt/β-catenin, AURK, BET for BAZ1B-dependent tumors, RARP for *KMT2C* and *KMT2D* mutant tumors, and RAS for *RAS* mutant tumors. ARID1A-deficient tumors may benefit from targeting ATR checkpoint activity, the non-catalytic role of EZH2, the PI3K/Akt pathway, PARP, and HDAC inhibitors

In this section, we summarize the findings from previous studies and explore potential therapeutic strategies for patients with MSI-H/dMMR GC. These strategies include a dual immune checkpoint blockade, combining PD-1/PD-L1 Abs with molecular targeted agents, and targeting vulnerabilities with selective molecule inhibition (Fig. 2).

### Immune therapy in a palliative setting

Several treatment strategies have been explored to transform immunologically “cold” tumors with poor immune activation into “hot” tumors with robust immune infiltration. The evolving landscape of immunotherapy in MSI-H/dMMR GC highlights the importance of combination approaches targeting multiple immune checkpoints and pathways to overcome resistance mechanisms. Combination therapies of anti-PD-1/PD-L1 Ab with other immune-modulating treatments, including other ICIs, angiogenetic inhibitors, and molecular targeted agents, present a promising avenue for treating MSI-H/dMMR GC by enhancing treatment efficacy through potential synergistic effects (Tables 4 and 5 and Fig. 2).

**Table 4:** Potent targeted molecules for MSI-H/dMMR GC patients

Molecules	Special comments
Immune checkpoint molecules	
CTLA-4	CTLA-4, exclusively expressed on T cells, acts early as a negative regulator of T cell priming by competitively binding to costimulatory molecules CD80/CD86 against CD28 located on antigen-presenting cells [6, 257]. As PD-1/PD-L1 inhibits antitumor T cell responses in later stages, dual inhibitors may synergistically enhance the antitumor immune response by blocking complementary mechanisms
TIGIT	TIGIT is highly expressed on activated T cells, NK cells, and regulatory T cells (Tregs), which bind to CD155 with high affinity and compete with its activating counter-receptor CD226, resulting in immune suppression [258]. TIGIT is often co-expressed with PD-1 on tumor-infiltrating CD8 +T cells in the TME, representing a potential mediator of immune evasion and ICI resistance [258]
TIM3	TIM3, expressed on innate immune cells, is a crucial checkpoint that can induce T cell apoptosis and exhaust type 1 CD4+ T cells and type 1 CD8+ T cells by binding to its ligand, glycan-binding protein-9, expressed on cancer cells [184]. Preclinical blockade of TIM3 restored the functions of TILs [259], overcame resistance to anti-PD-1 Ab [260], and synergistically promoted effects when combined with anti-PD-1 Ab [261]
LAG-3	LAG-3 is a cell-surface molecule expressed on immune cells that has high-affinity binding to MHC class II, leading to the activation of Tregs and the suppression of DCs and CD8 +T cells. LAG-3 and PD-1 are distinct inhibitory immune checkpoints that are often co-expressed on TILs, contributing to T cell exhaustion [262]. Preclinical models showed synergistic antitumor activity with a dual blockade of LAG-3 and PD-1 [263]. In the phase III RELATIVITY-047 trial, the combination of anti-LAG-3 Ab relatlimab with nivolumab provided a greater PFS benefit than nivolumab alone in advanced melanoma [264]. In MSI-H/dMMR GC, the frequent expression of TIM3 and LAG-3 in tumor-infiltrating immune cells [183] suggests that a dual blockade of PD-1 and LAG-3 could be effective for MSI-H/dMMR GC
OX40	Stimulatory immune checkpoints, such as OX40, can activate T cell functions [265]. OX40, expressed on all T cell subsets, interacts with OX40L on antigen-presenting cells, stimulating T cell responses, memory T cell expansion, DC activation, Treg depletion, and cytokine production [266]
RTK/RAS/MAPK and PI3K	
ERBB2/RAS	The success in targeting KRAS G12C in CRC [222] and HER2 in lung cancer [223] offers hope for MSI-H/dMMR GC harboring these alterations
PI3K pathway	The PI3K/PTEN/Akt pathway is more frequently dysregulated in MSI-H GC than MSS GC, particularly through mutations of *PIK3CA*, phosphoinositide-3-kinase regulatory subunit 1 (*PIK3R1*), and *PTEN* (Table 2) [2, 3, 20, 23, 57, 67, 92, 107, 119, 133, 247], and the activated signaling plays a significant role in the pathogenesis of MSI-H tumors [177] and potentially predicts primary resistance to ICIs in MSI-H/dMMR GC [92, 180]. Conversely, MSI-H/dMMR GC with mutated genes in the PI3K pathway showed sensitivity to inhibitors of the PI3K pathway [92]
VEGF/VEGFR	Angiogenesis contributes to the progression of carcinogenesis and metastasis by providing growth factors, nutrition, and an oxygen supply [267]. VEGF and its receptor VEGFR are key mediators responsible for angiogenesis. The dependency on angiogenesis is likely to be lower in MSI-H GC than in MSS GC [268]. However, in the transcriptomic analysis of RNA-seq in patients with MSI-H/dMMR gastrointestinal tumors, including GC, treated with PD-1 blockade, VEGF-A was significantly correlated with enriched pathways in non-responders to PD-1 blockade [179]. Lenvatinib targets VEGFR1-3, platelet-derived growth factor receptor (PDGFR) α, fibroblast growth factor receptor (FGFR), c-KIT, and RET tyrosine kinases. A systematic review showed more promising antitumor activity in solid cancers with lenvatinib plus pembrolizumab than with either lenvatinib or pembrolizumab alone [269].
Cell adhesion and proliferation	
Wnt/β-catenin pathway	The Wnt/β-catenin pathway is aberrantly activated in MSI-H/dMMR GC. Therapeutic strategies targeting the Wnt/β-catenin pathway have been explored [270], and preclinical studies suggest that *RNF43* mutations may serve as a predictive biomarker for inhibitors of Wnt/β-catenin signaling in pancreatic adenocarcinoma [271]. Activated Wnt/β-catenin pathway also contributes to immune evasion and resistance to immunotherapies in GC 16, and inhibitors of the Wnt/β-catenin pathway could be promising combination partners for ICIs [272]. Considering the predominant mutation of *RNF43* in MSI-H GC [232], targeting the Wnt/β-catenin signaling may have a potential antitumor response and enhance the efficacy of immunotherapy in MSI-H/dMMR GC
TGFβ pathway	The TGF-β signaling pathway plays a crucial immunosuppressive role in the TME by limiting T cell infiltration in tumors [273] and promoting the development of protumor phenotype tumor-associated neutrophils [274]. Despite frequent mutations in *ACVR2A* and *TGFBR2* leading to downregulation of the TGF-β signaling pathway in most MSI-H/dMMR GCs and CRCs due to instability of their MSs [32–34, 67, 68, 92, 107, 122, 124–126], the TGF-β-dependent stromal subset within MSI-H/dMMR CRCs exhibits reduced CD8 +TILs and increased expression of transcriptional signatures associated with ICI resistance [275]. Activation of the TGF-β pathway in the TME was associated with non-responders to the blocking of PD-1 in MSI-H/dMMR gastrointestinal tumors, including GS [179]. Therefore, concurrent inhibition of TGF-β signaling and immune checkpoint molecules may represent a promising therapeutic approach for patients with MSI-H/dMMR GCs
Chromatin regulation	
KMT2C/KMT2D	In MSI-H/dMMR GC, *KMT2C* and *KMT2D* mutations are linked to DNA repair, making them potential targets for treatment with PARP inhibitors. In addition, mutations in the *KMT2C* and *KMT2D* genes are promising biomarkers for immunotherapy [134]
ARID1A	Aberration of ARID1A promotes increased reliance on ATR checkpoint activity, and ATR inhibitors can act as synthetic lethal therapy for ARID1A-deficient tumors [209]. In large-scale drug sensitivity screening using GC organoids, both the ARID1A mutation and MSI-H displayed greater sensitivity to ATR inhibitors [276]
EZH2	EZH2 is a catalytic subunit of the polycomb repressive complex that can epigenetically alter gene expression via histone methyltransferase [277]. The MSI subtype is characterized by activation of EZH2, suggesting that further silencing of gene expression by EZH2 may play a role in the progression of the MSI subtype [42]. As ARID1A and EZH2 are antagonistic in regulating the PI3K/Akt signaling pathway via phosphoinositide-3-kinase interacting protein 1 (PIK3IP1) at a functional level [277, 278], blockade of EZH2 in ARID1A-deficient GC may suppress PI3K/Akt signaling and lead to an antitumor effect [278]
DNA-damage response	
PRKDC	Targeting components of DDR pathways has emerged as a therapeutic strategy [242, 279]. The DDR pathway may contribute to the efficacy of ICIs through an increased mutation load and neoantigen burden due to the loss of normal DNA repair function [280]. Protein kinase, DNA-activated, catalytic polypeptide (*PRKDC*) mutation was significantly associated with a high expression of inhibitory immune checkpoints (PD-L1, TIM3, and LAG-3) in GC [130]. A preclinical model showed that knockout PRKDC enhanced the efficacy of anti-PD-L1 Ab, and that most patients whose tumors harbored *PRKDC* mutations responded to immunotherapy [130]. Thus, the DDR pathway and immune responses are connected and potentially synergistic, and combined treatment with ICI and DDR inhibitors may have the potential to reinforce antitumor immune activity in MSI-H/dMMR GC
AURK	AURK plays critical roles not only during mitosis but also in various non-mitotic functions, including the DNA damage response
Others	
Nedd8	Deficient MMR systems lead to a hypermutator phenotype, resulting in proteome instability and an abundance of misfolded protein aggregates. To compensate, dMMR cells rely on the Nedd8-mediated degradation pathway to clear misfolded proteins [281]. A novel signature-guided therapy algorithm identified the Nedd8-activating enzyme inhibitor in MSI-H tumors, including GS. Blocking the Nedd8 clearance pathway causes the accumulation of misfolded protein aggregates, inducing immunogenic cell death and cytotoxic T cell recruitment in dMMR tumors. Combining Nedd8-activating enzyme inhibitor treatment with PD-1 inhibition synergistically improved the antitumor response over treatment with either therapy alone in dMMR tumors [281]. Thus, proteome instability may be a vulnerability in MSI-H/dMMR tumors
WRN	WRN is a RecQ enzyme that plays a crucial role in genome maintenance
BAZ1B	BAZ1B, an atypical tyrosine–protein kinase, acts as a chromatin remodeler and contains a bromodomain

**Table 5:** Ongoing clinical trials of molecular targeted agents in MSI-H/dMMR gastric cancer

Trials	Population	Target	Inhibitor	Agents	Phase	Treatment	Primary endpoint
Perioperative setting							
NCT04744649 (NICE)	MSI-H/dMMR in an exploratory group	PD-1	Ab	Toripalimab (Tori)	II	NAC Tori + chemo	MPR
NCT03421288 (DANTE)	All	PD-L1	Ab	Atezolizumab (Atezo)	II/III	Periope chemo ± Atezo	EFS
NCT04592913 (MATTERHORN)	All	PD-L1	Ab	Durvalumab (Durva)	III	Periope chemo ± Durva	EFS
NCT04139135	All	PD-1	Ab	Serplulimab (Ser)	III	Periope chemo ± Ser	EFS
NCT04795661 (IMHOTEP)	MSI-H/dMMR	PD-1	Ab	Pembrolizumab (Pembro)	II	Periope Pembro	pCR
NCT03257163	EBV, MSI-H, or PD-L1 positive	PD-1	Ab	Pembrolizumab (Pembro)	II	Periope Pembro and adj CRT	RFS
NCT04556253	MSI-H/dMMR	PD-L1 / CTLA-4	BiAb	AK104	II	Periope AK104	pCR
NCT04817826(INFINITY)	MSI-H/dMMR	PD-L1 / CTLA-4	Ab	Durvalumab (Durva) and Tremelimumab (Treme)	II	NAC or definitive Durva + Treme	pCR in NAC cohort 1 and 2-year CR rate in definitive cohort 2
NCT05994456	MSI-H/dMMR	PD-1	Ab	Toripalimab (Tori)	II	NAC Tori	pCR
NCT05836584(ECOG-ACRIN)	MSI-H/dMMR	PD-L1	Ab	Atezolizumab (Atezo)	II	Periope Atezo ± chemo	EFS
NCT05468138	MSI-H/dMMR	PD-1	Ab	Sintilimab (Sin)	II	Adj Sin vs. chemo vs. observation	3-year DFS
NCT05769725	EBV, MSI-H, or PD-L1 positive	PD-1	Ab	Serplulimab (Ser)	II	Adj chemo ± Ser	DFS
Metastatic setting							
NCT05177133 (AuspiCiOus)	MSI-H/dMMR solid cancer	PD-1	Ab	Retifanlimab (Reti)	II	Chemo for two cycles followed by Reti	Effect on the TME
NCT05483400	MSI-H solid cancer	TIGIT	Ab	Tiragolumab (Tirago)	II	Tirago + Atezo	BOR
NCT05144854 (ONO-4538-113)	All	PD-L1/CTLA-4	Ab	Nivolumab (Nivo) and ipilimumab (Ipi)	III	Chemo ± Nivo plus Ipi	OS
NCT04198766	MSI-H/dMMR solid cancer	OX40	Ab	INBRX-106	I/II	INBRX-106 +Pembro	Safety
NCT03894618	MSI-H/dMMR solid cancer	PD-1/OX40L	ARC	SL-279252	I	SL-279252	MTD
NCT04739202 (IMMUNOGAST)	EBV or MSI-H	AKT	SMI	Ipatasertib (Ipat)	II	Ipat + Atezo	ORR
NCT03407976	MSI-H/dMMR solid cancer or GC	VEGFR2	SMI	Apatinib (Apa)	I/II	Apa + Pembro	ORR
NCT04662710 (LEAP-015)	All	VEGFR 1–3	SMI	Lenvatinib (Lenva)	III	Chemo ± Lenva plus Pembro	OS and PFS
NCT05867121	GC cohort	TGF-β1	Ab	RO7496353 (RO)	Ib	RO + Nivo + Chemo	Safety
NCT05838768	MSI-H/dMMR solid cancer	WRN	SMI	HRO761	I	HRO761 + tislelizumab or CPT-11	Safety

Lenvatinib targets VEGFR1-3, platelet-derived growth factor receptor (PDGFR) α, fibroblast growth factor receptor (FGFR), c-KIT, and RET

*NCT number* ClinicalTrials.gov Identifier, *EBV* Epstein–Barr virus, *Ab* antibody, *BiAb* bispecific antibody, *SMI* small molecule inhibitor, *ARC* agonist redirected checkpoint, *CPT-11* irinotecan, *Periope chemo* perioperative chemotherapy, *NAC* neoadjuvant chemotherapy, *CRT* chemoradiotherapy, *Adj* adjuvant chemotherapy, *MPR* major pathologic response, *EFS* event-free survival, *pCR* pathological complete response, *DFS* disease-free survival, *TME* tumor microenvironment, *BOR* best overall response rate, *MTD* maximum tolerated dose, *ORR* overall response rate, *OS* overall survival, *PFS* progression-free survival

#### Dual inhibition of immune checkpoint molecules

The PD-1/PD-L1 interaction is just one of several immune checkpoint pathways regulating T cell activation in the TME. Other molecules, such as anti-cytotoxic T-lymphocyte associated antigen-4 (CTLA-4), T cell immunoreceptor with Ig and ITIM domains (TIGIT), T cell immunoglobulin mucin receptor 3 (TIM3), and lymphocyte activation gene 3 protein (LAG-3), are also overexpressed in various immune cells and act as inhibitory immune checkpoint modulators in GC (Table 4) [130, 183]. These inhibitory immune checkpoints may induce tumors to lose immunogenicity, contributing to reduced sensitivity to immunotherapy [6, 137, 184].

The most promising strategy is the dual blockade of PD-1 and CTLA-4. The phase I/II CheckMate-032 trial assessed the efficacy and safety of anti-PD-1 Ab nivolumab monotherapy or two different schedules of nivolumab plus anti-CTLA-4 Ab ipilimumab in patients with chemotherapy-refractory GC [14]. Patients were randomly assigned to one of the following treatment groups: nivolumab at 3 mg/kg (NIVO3); nivolumab at 1 mg/kg plus ipilimumab at 3 mg/kg (NIVO1 + IPI3) every 3 weeks for four cycles; or nivolumab at 3 mg/kg plus ipilimumab at 1 mg/kg (NIVO3 + IPI1) every 3 weeks for four cycles. All combination regimens were followed by NIVO3 every 2 weeks until disease progression or unacceptable toxicity. The NIVO1 + IPI3 group showed the highest ORR and 12-month PFS rate among the three groups, despite having the highest frequency of treatment-related adverse events. In a subset of MSI-H GC patients, treatment with nivolumab plus ipilimumab showed comparable outcomes in the ORR and 18-month PFS rates in the NIVO1 + IPI3 and NIVO3 + IPI1 groups. This approach demonstrated a tendency toward improved ORR (50% vs. 29%) and 18-month PFS rates (50% vs. 29%) compared to nivolumab monotherapy. In a phase III CheckMate 649 trial, the combination of nivolumab plus ipilimumab, as well as nivolumab plus chemotherapy, versus chemotherapy alone was evaluated in the first-line setting for GC [71]. Based on the results of the CheckMate-032 trial, the NIVO1 + IPI3 schedule was adopted. A limited number of patients had the MSI-H phenotype, with 11 patients for nivolumab plus ipilimumab and 10 patients for chemotherapy alone. Although the treatment group with nivolumab plus ipilimumab was discontinued early due to unacceptable toxicities, nivolumab plus ipilimumab showed a higher ORR (70% vs. 57%) and a longer median OS (HR, 0.28; 95% CI 0.08–0.92) compared with chemotherapy alone in patients with MSI-H GC. The group with nivolumab plus ipilimumab also demonstrated a more favorable ORR (70% vs. 55%) and an estimated HR for OS (0.28 vs. 0.38) than the group with nivolumab plus chemotherapy in a small subset of patients with 29 patients with MSI-H GC. In a single-arm phase II NO LIMIT trial of first-line nivolumab plus ipilimumab for MSI-H GC [185], low-dose ipilimumab at 1 mg/kg was chosen to reduce toxicity, considering the higher toxicity of the NIVO1 + IPI3 schedule in the CheckMate-032 trial. The ORR was 62.1%, with a clinical complete response (CR) rate of 10.3% and a DCR of 79.3%. The median PFS was 13.8 months, and the 12-month OS rate was 80%, suggesting a potential chemotherapy-free option for MSI-H GC patients. However, treatment-related adverse events led to discontinuation in 44.8% of the patients, indicating that further development of this regimen may require adjustments for improved feasibility. A phase III ONO-4538-113 trial (NCT05144854) to compare the efficacy and safety of nivolumab plus ipilimumab in combination with chemotherapy versus chemotherapy alone as a first-line treatment for patients with GC is currently underway.

Targeting various immune checkpoints, such as TIGIT, TIM3, LAG-3, and OX40, as well as CTLA-4, has shown promising results in preclinical and clinical studies in various types of tumors, suggesting their potential as therapeutic strategies in cancer treatment (Table 4). The efficacy of dual blockade targeting TIGIT and PD-1 is being assessed in the phase III STAR-221 trial (NCT05568095) in the first-line setting for GC, as well as in a cohort of metastatic MSI-H tumors from the phase II basket TIRACAN trial (NCT05483400) (Table 5). The efficacy of OX40 agonist Ab is currently under investigation in a phase I/II trials in certain solid tumors, including MSI-H/dMMR tumors (NCT04198766 and NCT03894618).

#### Combination with angiogenesis‑ or other molecular‑targeted agents

Vascular endothelial growth factor (VEGF) and its receptor (VEGFR) signaling pathway induces immunosuppressive effects via the downregulation of MHC expression, the activation of inhibitory immune checkpoint molecules, and the inhibition of TILs and DC differentiation [6, 186]. The most compatible partners of ICIs have been found to be anti-angiogenic inhibitors and platinum chemotherapy in a cross-sectional study of 98 clinical trials that included 24,915 patients [187], supporting combination treatment utilizing an anti-angiogenic inhibitor with ICIs (Table 4). Currently, several trials evaluating the efficacy of combining ICIs with anti-angiogenic inhibitors are ongoing in GC (NCT03407976 and NCT04662710) (Table 5). The understanding of angiogenesis in the TME may contribute to overcoming primary resistance to ICI in MSI-H/dMMR GC.

The PI3K/PTEN/v-akt murine thymoma viral oncogene homolog (Akt)/mTOR pathway is more frequently dysregulated in MSI-H GC than MSS GC (Table 2), and the activated signaling potentially predicts primary resistance to ICIs in MSI-H/dMMR GC [92, 180]. Conversely, MSI-H/dMMR GC with mutated genes in the PI3K pathway showed sensitivity to inhibitors of the PI3K pathway [92]. Simultaneous inhibition of the PI3K pathway may overcome resistance to ICIs as an immunotherapeutic adjunct in populations with activated PI3K pathways. As the H1047R mutation in exon 20 of *PIK3CA* is a common alteration in MSI-H/dMMR GC [3], the selective PI3Kα H1047R inhibitor, such as LOXO-783, may have a therapeutic effect [188]. Ipatasertib is a highly selective ATP-competitive pan-Akt inhibitor targeting phosphorylated Akt1-3. In a phase II trial (NCT04739202), patients with GC positive for EBV or MSI-H will receive treatment with atezolizumab and ipatasertib (Table 5).

A deficient MMR system causes cells to accumulate shared immunogenic frameshift peptide neoantigens [189]. In a phase I/IIa trial evaluating the safety and immunogenicity of a frameshift peptide neoantigen-based vaccine in patients with MSI/dMMR CRC, this approach showed a promising novel strategy for the treatment and prevention of dMMR tumors [190]. Molecules in the TGF-β, Wnt/β-catenin, DDR, and NEDD8 ubiquitin-like modifier (Nedd8)-mediated degradation pathways are also reported as potent therapeutic targets (Table 4). An in-depth understanding of the functional roles and molecular mechanisms of MSI-H/dMMR GC is crucial for developing targeted therapies.

### Immune therapy in a perioperative setting

Several trials have reported the promising treatment efficacy of neoadjuvant ICI in lung cancer, melanoma, bladder cancer, and CRC [191–195], indicating that immunotherapy may be highly effective in patients with early-stage cancer. Furthermore, neoadjuvant anti-PD-1 Ab dostarlimab monotherapy has demonstrated highly impressive results in that all 12 patients had CR and consequently avoided chemoradiotherapy and surgery in locally advanced dMMR rectal cancer [196]. The encouraging findings raise the hypothesis that the clinical efficacy of ICI treatment in early-stage MSI-H/dMMR tumors, prior to the emergence of immune evasion mechanisms facilitating metastatic dissemination, may surpass that in metastatic stages, prompting an exploration of ICI treatment in resectable MSI-H/dMMR GC (Table 5).

The integration of ICI into the perioperative chemotherapy has not been established across all populations of GC [77, 197]. However, in a meta-analysis of seven prospective phase I/II trials on ICI-based neoadjuvant therapy, 57 patients with MSI-H/dMMR GC had higher rates of pathological complete response (pCR) and major pathologic responses (MPR) compared to 244 patients with MSS/pMMR GC [198]. The potential benefits of ICI plus chemotherapy for MSI-H/dMMR GC have been observed in exploratory analyses of the phase III KEYNOTE-585 [77] and ongoing MATTERHORN (NCT04592913) [78] trials, as well as in recent phase II trials [74, 199–201]. Several trials are evaluating the efficacy of combining ICI with perioperative chemotherapy (NCT03421288, NCT04139135, NCT04592913, NCT04744649) (Table 5). Subgroup data derived from the MSI/MMR status in these trials would enhance the hypothesis that perioperative ICI treatment is beneficial for MSI-H/dMMR GC patients.

It is crucial to evaluate the need to incorporate cytotoxic agents into immunotherapy for an optimal treatment strategy. A phase II GERCOR NEONIPIGA trial evaluated the pathological response rate and safety of neoadjuvant nivolumab plus low-dose ipilimumab at 1 mg/kg for six cycles, followed by adjuvant nivolumab for 9 months in patients with resectable MSI-H/dMMR GC [202]. Of the 32 enrolled patients, 29 underwent curative surgery, with a pCR rate of 58.6%. The rate of pathological complete regression in the primary tumor for patients with MSI-H/dMMR was 66% in this trial using neoadjuvant ICI alone, comparable to the rate of 66% for patients with MSI-H/dMMR in the DANTE/IKF-s633 trial using ICI plus chemotherapy [74], prompting inquiries into the potential benefits of perioperative cytotoxic agents. At a median follow-up of 12 months, 30 (93.7%) patients remained alive without disease progression. It is noteworthy that three patients who did not undergo surgery achieved clinical CR and remained event-free. A phase II INFINITY trial (NCT04817826) is investigating dual ICI blockade using durvalumab plus anti-CTLA-4 Ab tremelimumab in the neoadjuvant (cohort 1) and potentially definitive (cohort 2) treatment for resectable MSI-H/dMMR GC [203]. In a cohort 1, 15 patients received a 12-week treatment followed by surgery, achieving a pCR rate of 60%. No disease relapses were observed in all patients with pCR. Non-operative management following the same regimen is being explored with cohort 2. Other ongoing phase II trials of perioperative treatment with ICI alone include NCT04795661, NCT03257163, NCT05994456, and NCT04556253 (Table 5). A direct comparison of perioperative ICI plus chemotherapy against perioperative ICI monotherapy is being evaluated in a randomized phase II ECOG-ACRIN trial (NCT05836584). These trials will provide proof-of-concept data for potentially omitting chemotherapy or surgery in selected patients after neoadjuvant immunotherapy.

Several preclinical studies have shown that neoadjuvant PD-1/PD-L1 blockade disrupts immunodominance and facilitates the early establishment of immunological memory following primary tumor resection, a phenomenon not observed in the adjuvant setting [204, 205]. This phenomenon contributes to the eradication of minimal residual disease and micro-metastases. Consequently, the efficacy of ICIs as an adjuvant therapy after surgery in improving outcomes for MSI-H/dMMR GC patients remains uncertain. Valuable insights are expected from the exploratory analysis of data from ICI-containing adjuvant trials, such as the phase III ATTRACTION-5 trial [197] and the phase III CheckMate-577 trial [206]. Currently, several phase II trials have investigated the treatment efficacy of adjuvant ICI (NCT05769725 and NCT05468138) (Table 5).

Although the limited sample size and short follow-up period hindered conclusive evidence, a growing number of impressive outcomes suggest a potential paradigm shift in approach to neoadjuvant immunotherapy or non-operative strategies for early-stage MSI-H/dMMR GC, emphasizing the importance of future dedicated clinical trials. Further research is required to determine the optimal regimen and duration for perioperative immunotherapy.

### Potent molecular‑targeted therapies

Therapeutic vulnerability has garnered considerable attention as a new hope for patients with MSI-H/dMMR GC (Table 4 and Fig. 2). DNA repair processes represent attractive synthetic lethal targets in MSI-H/dMMR tumors due to impaired DNA repair pathways, leading to a reliance on specific repair proteins. For example, the downregulation of KMT2C in bladder cancer leads to changes in the epigenetic status and expression of DDR and DNA repair genes, particularly affecting homologous recombination-mediated DNA double-strand break (DSB) repair. Thus, cancers with low KMT2C expression rely heavily on poly (ADP-ribose) polymerase (PARP) for DNA repair, and treatment with the PARP inhibitor leads to synthetic lethality [207]. In MSI-H/dMMR GC, *KMT2C* and *KMT2D* mutations are linked to DNA repair, making them potential targets for treatment with PARP inhibitors. The SWI/SNF complex is frequently mutated in MSI-H/dMMR GC, and therapeutic agents targeting SWI/SNF are emerging [208]. SWI/SNF-altered cancers may be sensitive to DNA-damage repair inhibitors and ICIs [133, 208].

As the aberration of ARID1A promotes increased reliance on ATR checkpoint activity caused by topoisomerase 2A and cell cycle defects, ATR inhibitors can act as synthetic lethal therapy for ARID1A-deficient tumors, both in vitro and in vivo [209]. ARID1A-deficient cancers are dependent on the non-catalytic role of the zeste 2 polycomb repressive complex 2 subunit (EZH2), promising therapeutic utility for ARID1A-deficient tumors [210]. Several preclinical studies have reported other crucial targets that induce synthetic lethality with ARID1A deficiency, such as PARP [211], ARID1B [212], the glutamate-cysteine ligase synthetase catalytic subunit [213], histone deacetylase 6 (HDAC6) [214], and BIRC5/Survivin [215]. Thus, inhibiting molecules that create the therapeutic vulnerability of ARID1A-deficient tumor cells may be of clinical importance.

In a CRISPR/dCas9 genome-wide screening of MSH2-deficient GC cells, the bromodomain adjacent to zinc finger domain 1B (BAZ1B) was identified as a synthetic lethal partner [216]. As both MSH2 and BAZ1B play roles in regulating the transcription of cell adhesion genes, MSH2-deficient GC cells can become dependent on BAZ1B, leading to synthetic lethality through the inhibition of the bromodomain and extraterminal motif (BET). This effect has also been observed in MSI-H GC cells. MSI-H/dMMR GCs show increased expression of mitotic network components, including aurora kinase (AURK) [2, 32]. AURK inhibitors were identified as potential candidate drugs for GCs with high immune activity, characterized by high TMB-H and MSI-H, based on the connectivity map database and gene set enrichment analysis [217].

Werner syndrome protein (WRN) is a RecQ enzyme that plays a crucial role in genome maintenance. Inhibition of WRN leads to DSBs, which selectively induce cell cycle arrest and apoptosis in MSI-H/dMMR tumors, including GC, due to their reliance on WRN’s helicase activity, unlike MSS/pMMR tumors [218]. Therefore, WRN represents a potential synthetic lethal vulnerability and a promising therapeutic target for MSI-H/dMMR tumors. HRO761, an allosteric WRN inhibitor, binds to the interface of the D1 and D2 helicase domains, rendering WRN inactive. This leads to WRN protein degradation and activates the DNA-damage response, resulting in tumor growth inhibition, specifically in MSI-H cell and patient-derived xenograft models [219]. A phase I trial is currently underway to evaluate the safety, tolerability, and preliminary antitumor activity of HRO761 alone or in combination with anti-PD-1 Ab tislelizumab or irinotecan in patients with MSI-H/dMMR solid tumors (NCT05838768) (Table 5).

MSI-H/dMMR GC typically lacks targetable amplifications. Notably, patients with *BRAF*-mutated MSI-H CRC had favorable outcomes with *BRAF*-targeted inhibitors in the phase III BEACON CRC trial [220], suggesting that molecular targeted therapy holds promise for MSI-H/dMMR GC. Considering the high incidence of Kirsten rat sarcoma viral oncogene homolog (*KRAS*) mutation in MSI-H/dMMR GC [2, 118, 221], the success in targeting KRAS G12C offers hope for developing allele-specific therapies for various mutant *RAS* alleles [222]. MSI-H/dMMR GC generally lacks targetable amplifications of HER2 but shows its frequent mutation [2]. In *HER2*-mutant non-small-cell lung cancer, an HER2 Ab-drug conjugate trastuzumab deruxtecan showed durable antitumor activity [223]. Loss of ARID1A leads to activation of the PI3K/Akt pathway via concurrent *PIK3CA* mutation and accelerated phosphorylation of Akt, and ARID1A-deficient GCs may be vulnerable to inhibitors of Akt or PIK3CA [114, 224].

The identification of a population vulnerable to specific molecular inhibition could lead to personalized molecular targeted medicine for MSI-H/dMMR GC patients (Fig. 2). In addition, assessing circulating tumor DNA (ctDNA) for intra- and inter-tumoral heterogeneity can help identify clonally altered genes in MSI-H/dMMR GC, guiding the selection of patients who may benefit from molecular targeted agents. As there is currently insufficient scientific evidence to establish these therapeutic strategies for MSI-H/MMR GC, further preclinical and clinical studies are needed for MSI-H/MMR GC.

## Conclusion

MSI-H/dMMR GC is a distinct subtype characterized by specific molecular features and clinical implications. Testing for MSI or MMR status should be a standard practice to guide treatment selection in GC patients. Immunotherapy has shown promise in treating metastatic MSI-H/dMMR GC, and neoadjuvant ICIs have implications for organ-sparing strategies. However, MSI-H/dMMR GC exhibits significant heterogeneity in terms of genomic, immunologic, and clinical outcomes, and a subset of patients treated with ICI exhibits primary resistance. Future research should focus on developing biomarker-driven treatment strategies, identifying novel therapeutic targets, and exploring synergistic therapeutic partners to improve prognostic outcomes in MSI-H/dMMR GC. A deeper understanding of the biology of MSI-H/dMMR GC could reveal a population vulnerable to specific molecular inhibition, potentially leading to the establishment of personalized medicine.

### Supplementary Information

Below is the link to the electronic supplementary material.

Supplementary file1 (DOCX 174 KB)

## Data Availability

N/A.
