# Supplementary material for: Potent therapeutic strategy in gastric cancer with microsatellite instability-high and/or deficient mismatch repair
Source: Gastric Cancer. 2024 Jun 26;27(5):907–31. doi: 10.1007/s10120-024-01523-4 (PMC11335850; doi:10.1007/s10120-024-01523-4)
Supplement: Supplementary file 1 — Supplementary file1 (DOCX 174 KB) [file 10120_2024_1523_MOESM1_ESM.docx]

**Supplementary Table 1.** Studies of cytotoxic chemotherapy for MSI-H/dMMR GC patients

| **Author/trial** | **Study** | **MSI-H/all (No. of pts)** | **MSI frequency (%)** | **Stage of disease** | **Therapy** | **Results in MSI-H population** | **Reference** |
| --- | --- | --- | --- | --- | --- | --- | --- |
| Hayden et al. | Retrospective | 21/101 | 20.8 | Resectable I-III | Without periope chemo | Tendency toward a favorable prognosis | ^1^ |
| Yamamoto et al. | Retrospective | 27/140 | 19.3 | I-IV | Without periope chemo | A better prognosis and an independent prognostic factor | ^2^ |
| Schneider et al. | Retrospective | 26/143 | 18.2 | Resectable I-IV | Without periope chemo | A better prognosis | ^3^ |
| Beghelli et al. | Retrospective | 83/510 | 16.3 | Resectable I-IV | Without periope chemo | A better prognosis only in stage II | ^4^ |
| Fang et al. | Retrospective | 25/214 | 11.7 | Resectable I-III | Without periope chemo | A better prognosis and an independent prognostic factor | ^5^ |
| Mathiak et al. | Retrospective | 34/452 | 7.5 | Resectable I-IV | Without periope chemo | A better prognosis | ^6^ |
| Shin et al. | Retrospective | 57/288 | 19.8 | Resectable I-III | Without periope chemo | Tendency toward a favorable prognosis | ^7^ |
| Dutch D1/D2 trial | Randomized (post hoc analysis) | 47/447 | 10.5 | Resectable I-IV | Without periope chemo | Tendency toward a favorable prognosis | ^8^ |
| Pereira et al. | Retrospective | 58/220 | 26.4 | Resectable I-IV | With or without periope chemo | A better prognosis | ^9^ |
| Vos et al. | Retrospective | 82/535 | 15.3 | Resectable II-IV | With or without periope chemo | A better prognosis and an independent prognostic factor. No benefit of periope chemo over surgery alone | ^10^ |
| Zhu et al. | Meta-analysis | 431/1976 | 21.8 | Resectable I-IV | With or without periope chemo | A better prognosis | ^11^ |
| Quaas et al. | pooled analysis | 115/1307 | 8.8 | Resectable I-IV | With or without periope chemo | A better prognosis in females, but not males | ^12^ |
| CRITICS trial | Randomized (post hoc analysis) | 27/454 | 5.9 | Resectable I-IV | Periope chemo | A better prognosis, but poor pathological response to periope chemo | ^8^ |
| Oh et al. | Retrospective | 100/838 | 11.9 | pT1N1 | With or without Adj | No benefit of Adj over surgery alone, and no impact on prognosis | ^13^ |
| Kim et al. | Retrospective | 105/1276 | 8.2 | Resectable II-III | With or without Adj | Detrimental effect of Adj over surgery alone | ^14^ |
| Marrelli et al. | Retrospective | 111/472 | 23.5 | Resectable I-IV | With or without Adj | A better prognosis in noncardiac intestinal type | ^15^ |
| Sohn et al. | Retrospective | 57/262 | 21.8 | Resectable I-IV | With or without Adj | Poorer prognosis than those with EBV subtype, but better than those with GS subtype, and moderate benefit of Adj | ^16^ |
| Kwon et al. | Retrospective | 37/394 | 9.4 | Resectable I-IV | With or without Adj | A better prognosis | ^17^ |
| Huang et al. | Retrospective | 116/1248 | 9.3 | Resectable I-IV | With or without Adj | No benefit of Adj over surgery alone, and tendency toward a favorable prognosis, especially in stage III | ^18^ |
| Kim et al. | Retrospective | cohort 1: 41/359 | 11.4 | Resectable IB-III | With or without Adj | No benefit of Adj, and tendency toward a favorable prognosis | ^19^ |
| Kim et al. | Retrospective | cohort 2: 162/162 | 100 | Resectable II-III | With or without Adj | Benefit of Adj | ^19^ |
| An et al. | Retrospective | 64/790 | 8.1 | Resectable II-III | With or without Adj | No impact on prognosis after recurrence, and better response to chemo after recurrence in MSI-H patients without Adj | ^20^ |
| Tsai et al. | Retrospective | 83/929 | 8.9 | Resectable II-IV | With or without Adj | A better prognosis in stage III, and no impact on prognosis between dMMR patients with or without Adj | ^21^ |
| Guan et al. | Retrospective | 196/890 | 22.0 | I-IV | With or without Adj | No benefit of Adj in stage II/III, and worse ORR and PFS in stage IV | ^22^ |
| Yang et al. | Retrospective | 54/226 | 23.9 | Resectable I-III | With or without Adj | No benefit of Adj over surgery alone | ^23^ |
| Choi et al. | Meta-analysis | 712/5,438 | 13.1 | Resectable I-IV | With or without Adj | A better prognosis | ^24^ |
| Nie et al. | Meta-analysis | 354/2186 | 16.2 | Resectable I-IV | With or without Adj | A better prognosis | ^25^ |
| Nie et al. | Meta-analysis | 501 | 100 | Resectable I-IV | With or without Adj | Benefit of Adj | ^25^ |
| Oki et al. | Retrospective | 22/240 | 9.2 | Resectable I-IV | 5-FU-based Adj | No impact on prognosis | ^26^ |
| An et al. | Retrospective | 170/1990 | 8.5 | Resectable I-IV | 5-FU-based Adj | no benefit of Adj in stage II–III, and no impact on prognosis | ^27^ |
| Sato et al. | Retrospective | 24/184 | 13.0 | Resectable II | Adj S-1 | A better prognosis | ^28^ |
| ITACA-S Trial | Randomized (post hoc analysis) | 23/256 | 9.0 | Resectable II-III | Adj 5-FU/LV vs. sequential FOLFIRI and CDDP + DTX | An independent prognostic factor | ^29^ |
| ARTIST Trial | Randomized (post hoc analysis) | 35/393 | 8.9 | Resectable I-IV | Adj XP or XPRT | Tendency toward a favorable prognosis | ^30^ |
| CLASSIC trial | Randomized (post hoc analysis) | 40/592 | 6.8 | Resectable II-III | Adj CAPOX vs. observation | No benefit of Adj and an independent prognostic factor | ^31, 32^ |
| MAGIC trial | Randomized (post hoc analysis) | 20/303 | 6.6 | Resectable II-III | periope chemo ECF vs. surgery alone | Detrimental effect of periope chemo over surgery alone, and poor pathological response | ^33^ |
| Pietrantonio et al. | Meta-analysis (MAGIC, CLASSIC, ARTIST and ITACA-S trials) | 121/1556 | 7.8 | Resectable I-IV | With or without periope chemo o | No benefit of Adj and an independent prognostic factor | ^34^ |
| Zhao et al. | Meta-analysis | 84/1174 | 7.2 | Resectable II-III | 5-FU-based Adj | No correlation between MSI status and efficacy of Adj, and tendency toward a favorable prognosis | ^35^ |
| Kim et al. | Retrospective | 88/881 | 10.0 | Resectable II-III | With or without adjuvant CRT | No benefit of adjuvant CRT, and an independent prognostic factor | ^36^ |
| Corso et al. | Prospective | 41/250 | 16.4 | Resectable I-IV | Without NAC | A better prognosis | ^37^ |
| Seo et al. | Retrospective | 27/328 | 8.2 | Resectable I-IV | Without NAC | No impact on prognosis | ^38^ |
| Kim et al. | Retrospective | 23/414 | 5.6 | Radical resection | Without NAC | Poor survival in intestinal type, but not in diffuse or overall GC | ^39^ |
| Cai et al. | Retrospective | 28/271 | 10.3 | Resectable I-IV | Without NAC | A better prognosis and an independent prognostic factor | ^40^ |
| Fan et al. | Retrospective | 66/187 | 35.3 | Resectable I-IV | Without NAC | A better prognosis | ^41^ |
| Polom et al. | Meta-analysis | 1718/18,612 | 9.2 | Resectable I-IV | Without NAC | A better prognosis | ^42^ |
| Kohlruss et al. | Retrospective | 74/760 | 9.7 | Resectable II-IV | With or without NAC | No impact on efficacy of NAC, but poor pathological response. A better prognosis irrespective of NAC. | ^43^ |
| Kohlruss et al. | Retrospective | 67/717 | 9.3 | Resectable I-IV | With or without NAC | A better prognosis in females with NAC and an independent prognostic factor | ^44^ |
| Cai et al. | Retrospective | 57/690 | 8.3 | Resectable III | With or without NAC | A better prognosis, and benefit of NAC despite of poor response to NAC | ^45^ |
| Kohlruss et al. | Retrospective | 56/612 | 9.2 | Resectable I-IV | With or without NAC | A better prognosis | ^46^ |
| Hashimoto et al. | Retrospective | 28/285 | 9.8 | Resectable I-IV | 5-FU-based NAC | Detrimental effect of NAC | ^47^ |
| do Nascimento et al. | Retrospective | 37/137 | 27.0 | Resectable | 5-FU-based NAC | No impact prognosis, but better prognosis in females. Poor pathological response of NAC. | ^48^ |
| Falchetti et al. | Retrospective | 27/159 | 17.0 | Radical resection | N/C | A better prognosis | ^49^ |
| Polom et al. | Retrospective | 102/386 | 26.4 | positive resection margin | N/C | A better prognosis | ^50^ |
| Zhang et al. | Retrospective | 57/567 | 10.1 | Resectable I-IV | N/C | A better prognosis | ^51^ |
| Ramos et al. | Retrospective | 60/287 | 20.9 | Resectable I-IV | N/C | A better prognosis and an independent prognostic factor | ^52^ |
| Tran-Minh et al. | Retrospective | 39/315 | 12.4 | I-IV | N/C | In patients with resected tumor without metastasis, the recurrence rate was low, but not an independent prognostic factor | ^53^ |
| Martinez-Ciarpaglini et al. | Retrospective | 45/246 | 18.3 | I-IV | N/C | A better prognosis in patients without periope chemo than those with periope chemo, and an independent prognostic factor in stage I-III. | ^54^ |
| KEYNOTE-061 | Randomized (post hoc analysis) | 12/296 | 4.1 | palliative setting | 2nd line PTX | In the chemotherapy arm, ORR was 12.5% vs. 16.7%, median PFS was 4.1 months vs. 3.5 months, and OS was 8.3 months vs. 8.1 months in all patients vs. MSI-H GC, respectively. | ^55^ |
| KEYNOTE-062 | Randomized (post hoc analysis) | 19/250 | 7.6 | palliative setting | 1st line CDDP + 5-FU or cape | In the chemotherapy arm, ORR was 37.2% vs. 36.8%, median PFS was 6.4 months vs. 6.6 months, and OS was 11.1 months vs. 8.5 months in all patients vs. MSI-H GC, respectively. | ^55^ |
| CheckMate 649 | Randomized (post hoc analysis) | 34/881 | 3.9 | palliative setting | 1st line CAPOX or FOLFOX | In the first-line chemotherapy arm, ORR was 46% vs. 39%, and OS was 11.5 months vs. 12.3 months in all patients vs. MSI-H GC, respectively. | ^56, 57^ |

This list included only studies with over 20 patients with MSI-H/dMMR.

**Abbreviations**: No of pts, number of patients; ORR, overall response rate; PFS, progression-free survival; OS, overall survival; periope chemo, perioperative chemotherapy; Adj, adjuvant chemotherapy; 5-FU, 5-fluorouracil; LV, leucovorin; FOLFIRI, 5-fluorouracil + LV + irinotecan; CDDP, cisplatin; DTX, docetaxel; XP, capecitabine + CDDP; RT, radiotherapy; CAPOX, capecitabine + oxaliplatin; ECF, epirubicin + CDDP + 5-FU; CRT, chemoradiotherapy; NAC, neoadjuvant chemotherapy; N/C, no certain information; PTX, paclitaxel; FOLFOX, 5-fluorouracil + leucovorin + oxaliplatin; GC, gastric cancer.

**References**

1. Hayden JD, Cawkwell L, Quirke P, et al. Prognostic significance of microsatellite instability in patients with gastric carcinoma. Eur J Cancer.

1997;33: 2342-2346.

2. Yamamoto H, Perez-Piteira J, Yoshida T, et al. Gastric cancers of the microsatellite mutator phenotype display characteristic genetic and clinical features. Gastroenterology. 1999;116: 1348-1357.

3. Schneider BG, Bravo JC, Roa JC, et al. Microsatellite instability, prognosis and metastasis in gastric cancers from a low-risk population. Int J Cancer. 2000;89: 444-452.

4. Beghelli S, de Manzoni G, Barbi S, et al. Microsatellite instability in gastric cancer is associated with better prognosis in only stage II cancers. Surgery. 2006;139: 347-356.

5. Fang WL, Chang SC, Lan YT, et al. Microsatellite instability is associated with a better prognosis for gastric cancer patients after curative surgery. World J Surg. 2012;36: 2131-2138.

6. Mathiak M, Warneke VS, Behrens HM, et al. Clinicopathologic Characteristics of Microsatellite Instable Gastric Carcinomas Revisited: Urgent Need for Standardization. Appl Immunohistochem Mol Morphol. 2017;25: 12-24.

7. Shin SJ, Kim SY, Choi YY, et al. Mismatch Repair Status of Gastric Cancer and Its Association with the Local and Systemic Immune Response. Oncologist. 2019;24: e835-e844.

8. Biesma HD, Soeratram TTD, Sikorska K, et al. Response to neoadjuvant chemotherapy and survival in molecular subtypes of resectable gastric cancer: a post hoc analysis of the D1/D2 and CRITICS trials. Gastric Cancer. 2022;25: 640-651.

9. Pereira MA, Ramos M, Faraj SF, et al. Clinicopathological and prognostic features of Epstein-Barr virus infection, microsatellite instability, and PD-L1 expression in gastric cancer. J Surg Oncol. 2018;117: 829-839.

10. Vos EL, Maron SB, Krell RW, et al. Survival of Locally Advanced MSI-high Gastric Cancer Patients Treated with Perioperative Chemotherapy: A Retrospective Cohort Study. Ann Surg. 2022.

11. Zhu L, Li Z, Wang Y, Zhang C, Liu Y, Qu X. Microsatellite instability and survival in gastric cancer: A systematic review and meta-analysis. Mol Clin Oncol. 2015;3: 699-705.

12. Quaas A, Biesma HD, Wagner AD, et al. Microsatellite instability and sex differences in resectable gastric cancer - A pooled analysis of three European cohorts. Eur J Cancer. 2022;173: 95-104.

13. Oh N, Kim H, Kim KM, et al. Microsatellite Instability and Effectiveness of Adjuvant Treatment in pT1N1 Gastric Cancer: A Multicohort Study. Ann Surg Oncol. 2021;28: 8908-8915.

14. Kim SY, Choi YY, An JY, et al. The benefit of microsatellite instability is attenuated by chemotherapy in stage II and stage III gastric cancer: Results from a large cohort with subgroup analyses. Int J Cancer. 2015;137: 819-825.

15. Marrelli D, Polom K, Pascale V, et al. Strong Prognostic Value of Microsatellite Instability in Intestinal Type Non-cardia Gastric Cancer. Ann Surg Oncol. 2016;23: 943-950.

16. Sohn BH, Hwang JE, Jang HJ, et al. Clinical Significance of Four Molecular Subtypes of Gastric Cancer Identified by The Cancer Genome Atlas Project. Clin Cancer Res. 2017.

17. Kwon MJ, Kim KC, Nam ES, et al. Programmed death ligand-1 and MET co-expression is a poor prognostic factor in gastric cancers after resection. Oncotarget. 2017;8: 82399-82414.

18. Huang SC, Ng KF, Yeh TS, et al. Subtraction of Epstein-Barr virus and microsatellite instability genotypes from the Lauren histotypes: Combined molecular and histologic subtyping with clinicopathological and prognostic significance validated in a cohort of 1,248 cases. Int J Cancer. 2019;145: 3218-3230.

19. Kim JW, Cho SY, Chae J, et al. Adjuvant Chemotherapy in Microsatellite Instability-High Gastric Cancer. Cancer Res Treat. 2020;52: 1178-1187.

20. An JY, Choi YY, Lee J, et al. A Multi-cohort Study of the Prognostic Significance of Microsatellite Instability or Mismatch Repair Status after Recurrence of Resectable Gastric Cancer. Cancer Res Treat. 2020;52: 1153-1161.

21. Tsai CY, Lin TA, Huang SC, et al. Is Adjuvant Chemotherapy Necessary for Patients with Deficient Mismatch Repair Gastric Cancer?-Autophagy Inhibition Matches the Mismatched. Oncologist. 2020;25: e1021-e1030.

22. Guan WL, Ma Y, Cui YH, et al. The Impact of Mismatch Repair Status on Prognosis of Patients With Gastric Cancer: A Multicenter Analysis. Front Oncol. 2021;11: 712760.

23. Yang Y, Shi Z, Bai R, Hu W. Heterogeneity of MSI-H gastric cancer identifies a subtype with worse survival. J Med Genet. 2021;58: 12-19.

24. Choi YY, Bae JM, An JY, et al. Is microsatellite instability a prognostic marker in gastric cancer? A systematic review with meta-analysis. J Surg Oncol. 2014;110: 129-135.

25. Nie RC, Chen GM, Yuan SQ, et al. Adjuvant Chemotherapy for Gastric Cancer Patients with Mismatch Repair Deficiency or Microsatellite Instability: Systematic Review and Meta-Analysis. Ann Surg Oncol. 2022;29: 2324-2331.

26. Oki E, Kakeji Y, Zhao Y, et al. Chemosensitivity and survival in gastric cancer patients with microsatellite instability. Ann Surg Oncol. 2009;16: 2510-2515.

27. An JY, Kim H, Cheong JH, Hyung WJ, Kim H, Noh SH. Microsatellite instability in sporadic gastric cancer: its prognostic role and guidance for 5-FU based chemotherapy after R0 resection. Int J Cancer. 2012;131: 505-511.

28. Sato C, Kawakami H, Tanaka R, et al. Survival impact of microsatellite instability in stage II gastric cancer patients who received S-1 adjuvant monotherapy after curative resection. Sci Rep. 2023;13: 10826.

29. Di Bartolomeo M, Morano F, Raimondi A, et al. Prognostic and Predictive Value of Microsatellite Instability, Inflammatory Reaction and PD-L1 in Gastric Cancer Patients Treated with Either Adjuvant 5-FU/LV or Sequential FOLFIRI Followed by Cisplatin and Docetaxel: A Translational Analysis from the ITACA-S Trial. Oncologist. 2020;25: e460-e468.

30. Miceli R, An J, Di Bartolomeo M, et al. Prognostic Impact of Microsatellite Instability in Asian Gastric Cancer Patients Enrolled in the ARTIST Trial. Oncology. 2019;97: 38-43.

31. Choi YY, Kim H, Yang H-K, et al. Clinical impact of microsatellite instability in patients with stage II and III gastric cancer: Results from the CLASSIC trial. Journal of Clinical Oncology. 2017;35: 4022-4022.

32. Choi YY, Kim H, Shin SJ, et al. Microsatellite Instability and Programmed Cell Death-Ligand 1 Expression in Stage II/III Gastric Cancer: Post Hoc Analysis of the CLASSIC Randomized Controlled study. Ann Surg. 2019;270: 309-316.

33. Smyth EC, Wotherspoon A, Peckitt C, et al. Mismatch Repair Deficiency, Microsatellite Instability, and Survival: An Exploratory Analysis of the Medical Research Council Adjuvant Gastric Infusional Chemotherapy (MAGIC) Trial. JAMA Oncol. 2017;3: 1197-1203.

34. Pietrantonio F, Miceli R, Raimondi A, et al. Individual Patient Data Meta-Analysis of the Value of Microsatellite Instability As a Biomarker in Gastric Cancer. J Clin Oncol. 2019;37: 3392-3400.

35. Zhao F, Yuan X, Ren D, et al. Predicting the Efficacy of 5-Fluorouracil-Based Adjuvant Chemotherapy in Gastric Cancer by Microsatellite Instability: A Meta-Analysis. J Environ Pathol Toxicol Oncol. 2019;38: 21-28.

36. Kim SM, An JY, Byeon SJ, et al. Prognostic value of mismatch repair deficiency in patients with advanced gastric cancer, treated by surgery and adjuvant 5-fluorouracil and leucovorin chemoradiotherapy. Eur J Surg Oncol. 2020;46: 189-194.

37. Corso G, Pedrazzani C, Marrelli D, Pascale V, Pinto E, Roviello F. Correlation of microsatellite instability at multiple loci with long-term survival in advanced gastric carcinoma. Arch Surg. 2009;144: 722-727.

38. Seo HM, Chang YS, Joo SH, et al. Clinicopathologic characteristics and outcomes of gastric cancers with the MSI-H phenotype. J Surg Oncol. 2009;99: 143-147.

39. Kim JY, Shin NR, Kim A, et al. Microsatellite instability status in gastric cancer: a reappraisal of its clinical significance and relationship with mucin phenotypes. Korean J Pathol. 2013;47: 28-35.

40. Cai L, Sun Y, Wang K, et al. The Better Survival of MSI Subtype Is Associated With the Oxidative Stress Related Pathways in Gastric Cancer. Front Oncol. 2020;10: 1269.

41. Fan JP, Qian J, Zhao YJ. The loss of PTEN expression and microsatellite stability (MSS) were predictors of unfavorable prognosis in gastric cancer (GC). Neoplasma. 2020;67: 1359-1366.

42. Polom K, Marano L, Marrelli D, et al. Meta-analysis of microsatellite instability in relation to clinicopathological characteristics and overall survival in gastric cancer. Br J Surg. 2018;105: 159-167.

43. Kohlruss M, Grosser B, Krenauer M, et al. Prognostic implication of molecular subtypes and response to neoadjuvant chemotherapy in 760 gastric carcinomas: role of Epstein-Barr virus infection and high- and low-microsatellite instability. J Pathol Clin Res. 2019;5: 227-239.

44. Kohlruss M, Ott K, Grosser B, et al. Sexual Difference Matters: Females with High Microsatellite Instability Show Increased Survival after Neoadjuvant Chemotherapy in Gastric Cancer. Cancers (Basel). 2021;13.

45. Cai Z, Rui W, Li S, et al. Microsatellite Status Affects Tumor Response and Survival in Patients Undergoing Neoadjuvant Chemotherapy for Clinical Stage III Gastric Cancer. Front Oncol. 2020;10: 614785.

46. Kohlruss M, Krenauer M, Grosser B, et al. Diverse 'just-right' levels of chromosomal instability and their clinical implications in neoadjuvant treated gastric cancer. Br J Cancer. 2021;125: 1621-1631.

47. Hashimoto T, Kurokawa Y, Takahashi T, et al. Predictive value of MLH1 and PD-L1 expression for prognosis and response to preoperative chemotherapy in gastric cancer. Gastric Cancer. 2019;22: 785-792.

48. do Nascimento CN, Mascarenhas-Lemos L, Silva JR, et al. EBV and MSI Status in Gastric Cancer: Does It Matter? Cancers (Basel). 2022;15.

49. Falchetti M, Saieva C, Lupi R, et al. Gastric cancer with high-level microsatellite instability: target gene mutations, clinicopathologic features, and long-term survival. Hum Pathol. 2008;39: 925-932.

50. Polom K, Marrelli D, Smyth EC, et al. The Role of Microsatellite Instability in Positive Margin Gastric Cancer Patients. Surg Innov. 2018;25: 99-104.

51. Zhang Q, Wang L, Ni S, et al. Clinicopathological features and prognostic value of mismatch repair protein deficiency in gastric cancer. Int J Clin Exp Pathol. 2018;11: 2579-2587.

52. Ramos M, Pereira MA, de Mello ES, et al. Gastric cancer molecular classification based on immunohistochemistry and in situ hybridization: Analysis in western patients after curative-intent surgery. World J Clin Oncol. 2021;12: 688-701.

53. Tran-Minh ML, Lehmann-Che J, Lambert J, et al. Prevalence and prognosis of microsatellite instability in oesogastric adenocarcinoma, NORDICAP 16-01. Clin Res Hepatol Gastroenterol. 2021;45: 101691.

54. Martinez-Ciarpaglini C, Fleitas-Kanonnikoff T, Gambardella V, et al. Assessing molecular subtypes of gastric cancer: microsatellite unstable and Epstein-Barr virus subtypes. Methods for detection and clinical and pathological implications. ESMO Open. 2019;4: e000470.

55. Chao J, Fuchs CS, Shitara K, et al. Assessment of Pembrolizumab Therapy for the Treatment of Microsatellite Instability-High Gastric or Gastroesophageal Junction Cancer Among Patients in the KEYNOTE-059, KEYNOTE-061, and KEYNOTE-062 Clinical Trials. JAMA Oncol. 2021;7: 895-902.

56. Shitara K, Ajani JA, Moehler M, et al. Nivolumab plus chemotherapy or ipilimumab in gastro-oesophageal cancer. Nature. 2022;603: 942-948.

57. Janjigian YY, Shitara K, Moehler MH, et al. Nivolumab (NIVO) plus chemotherapy (chemo) vs chemo as first-line (1L) treatment for advanced gastric cancer/gastroesophageal junction cancer/esophageal adenocarcinoma (GC/GEJC/EAC): 3-year follow-up from CheckMate 649. Journal of Clinical Oncology. 2023;41: 291-291.
